# Cellular response to spinal cord injury in regenerative and non-regenerative stages in *Xenopus laevis*

**DOI:** 10.1186/s13064-021-00152-2

**Published:** 2021-02-02

**Authors:** Gabriela Edwards-Faret, Karina González-Pinto, Arantxa Cebrián-Silla, Johany Peñailillo, José Manuel García-Verdugo, Juan Larraín

**Affiliations:** 1 Center for Aging and Regeneration, Departamento de Biología Celular y Molecular, Facultad de Ciencias Biológicas, Pontificia Universidad Católica de Chile, Alameda 340, Santiago, Chile; 2 Laboratorio de Neurobiología Comparada, Instituto Cavanilles, Universidad de Valencia, CIBERNED, 46980 Valencia, Spain

**Keywords:** Spinal cord, Regeneration, NSPCs, Neurogenesis, sox2, Gfap, Xenopus, Glial scar

## Abstract

**Background:**

The efficient regenerative abilities at larvae stages followed by a non-regenerative response after metamorphosis in froglets makes *Xenopus* an ideal model organism to understand the cellular responses leading to spinal cord regeneration.

**Methods:**

We compared the cellular response to spinal cord injury between the regenerative and non-regenerative stages of *Xenopus laevis*. For this analysis, we used electron microscopy, immunofluorescence and histological staining of the extracellular matrix. We generated two transgenic lines: i) the reporter line with the zebrafish GFAP regulatory regions driving the expression of EGFP, and ii) a cell specific inducible ablation line with the same GFAP regulatory regions. In addition, we used FACS to isolate EGFP^+^ cells for RNAseq analysis.

**Results:**

In regenerative stage animals, spinal cord regeneration triggers a rapid sealing of the injured stumps, followed by proliferation of cells lining the central canal, and formation of rosette-like structures in the ablation gap. In addition, the central canal is filled by cells with similar morphology to the cells lining the central canal, neurons, axons, and even synaptic structures. Regeneration is almost completed after 20 days post injury. In non-regenerative stage animals, mostly damaged tissue was observed, without clear closure of the stumps. The ablation gap was filled with fibroblast-like cells, and deposition of extracellular matrix components. No reconstruction of the spinal cord was observed even after 40 days post injury. Cellular markers analysis confirmed these histological differences, a transient increase of vimentin, fibronectin and collagen was detected in regenerative stages, contrary to a sustained accumulation of most of these markers, including chondroitin sulfate proteoglycans in the NR-stage.

The zebrafish GFAP transgenic line was validated, and we have demonstrated that is a very reliable and new tool to study the role of neural stem progenitor cells (NSPCs). RNASeq of GFAP::EGFP cells has allowed us to clearly demonstrate that indeed these cells are NSPCs. On the contrary, the GFAP::EGFP transgene is mainly expressed in astrocytes in non-regenerative stages. During regenerative stages, spinal cord injury activates proliferation of NSPCs, and we found that are mainly differentiated into neurons and glial cells. Specific ablation of these cells abolished proper regeneration, confirming that NSPCs cells are necessary for functional regeneration of the spinal cord.

**Conclusions:**

The cellular response to spinal cord injury in regenerative and non-regenerative stages is profoundly different between both stages. A key hallmark of the regenerative response is the activation of NSPCs, which massively proliferate, and are differentiated into neurons to reconstruct the spinal cord. Also very notably, no glial scar formation is observed in regenerative stages, but a transient, glial scar-like structure is formed in non-regenerative stage animals.

**Supplementary Information:**

The online version contains supplementary material available at 10.1186/s13064-021-00152-2.

## Introduction

Anatomical and functional regeneration of the spinal cord (SC) varies among the animal kingdom. While jawed and teleost fishes, urodele amphibians including salamanders and triton, lampreys and reptilians such as turtles, have very efficient regenerative capabilities [1–4]. Other species such as anuran amphibians, including *Xenopus laevis* (*X. laevis*) are able to regenerate the SC at larval stages, but this capacity is lost during metamorphosis [5]. On the contrary, mammals are not able to attain efficient regeneration after SC injury (SCI) producing long-lasting effects [6]. Comparing the cellular response to injury in regenerative and non-regenerative model organisms is important to understand which cells facilitate or impede spinal cord regeneration.

The mammalian adult SC central canal (CC) mainly contains ependymal cells astrocytes [7] and some cerebral fluid contacting neurons [8]. The presence of neural stem progenitor cells (NSPCs) has been demonstrated by in vitro experiments based on their ability to form neurospheres [9], and are mainly derived from cells expressing FoxJ1, Nestin or Glial fibrillary acidic protein (GFAP) [10–12]. NSPCs form neurons in vitro or when transplanted into a neurogenic environment, but not in the SC [13]. In rodents, spinal cord injury (SCI) generates more neurospheres, and nestin expression levels are increased, suggesting an activation of NSPCs [12, 14, 15]. Despite this, most activated NSPCs cells are fated to astrocytes, and formation of new neurons is not observed in vivo [10]. In spite of neurosphere formation, in vivo proliferation of NSPCs is rare, and just a very low proliferative capacity is present in dorsal and ventral uni- and Bi-ciliated ependymal cells [16].

Furthermore, following SCI a fibrotic and glial scar in the lesion site is formed by microglia, astrocytes, inflammatory cells, meningeal fibroblasts and pericytes together with an abundant extracellular matrix (ECM) containing Chondroitin Sulfate Proteoglycans (CSPGs) and Collagen [17, 18]. The main function of this scar is to contain the inflammatory response, protecting the spinal cord from further damage [19–21]. In addition, the scar blocks axonal growth, inhibiting spinal cord regeneration [22]. Although, recent work challenged this dogma and disproved that astrocytes were the main CSPGs producers, instead, astrocytes secretes neuronal growth factors essential for neuronal survival, but, other cell types produce CSPGs which lead to the lack of regeneration [23, 24].

A very different cellular response is observed in regenerative model organisms. In zebrafish and salamanders, the CC of the SC is mainly composed of ependymo-radial glia (ERG) cells. These cells are reminiscent of embryonic radial-glial, and express GFAP and Sox2, both markers of NSPCs [25–27]. ERG have different functions after SCI, including a role as neural progenitors, and the generation of a permissive substrate for axonal regeneration [27]. In both, zebrafish and salamanders, injury activates massive proliferation of ERG cells, and expression of Sox2 is required for activation of cell division [28, 29]. In fish, ERG cells are neural progenitors and can generate motoneurons and interneurons in response to injury [30–34]. Cell fate experiments showed that most of the new neurons are derived from a population of GFAP^+^ ERG cells [35].

In addition, ERG can also provide a permissive environment for axon growth in the adult fish. Processes from GFAP^+^ ERG cells elongate into the injury site connecting the two ends of the injured spinal cord making a “glial bridge” that could provide a permissive substrate for axon regeneration [35–38]. Interestingly, in zebrafish larvae, axons grow into the injure site before the glial bridge is formed suggesting that at this stage an “axonal bridging” between the two spinal cord stumps is established [27, 39].

*X. laevis* provides a unique experimental paradigm to compare regenerative and non-regenerative responses in the same specie [5, 40]. Pre-metamorphosis stages (NF stage 48–54) show a very efficient SC regeneration and are considered regenerative stages (R-stage). This ability is lost during metamorphosis (NF stage 66), and post-metamorphic animals including froglets are unable to regenerate the SC therefore are denominated as non-regenerative stages (NR-stages) [41–47]. At R-stages, most cells lining the CC have a radial glial morphology, are uniciliated, and express Sox2 [48]. While in NR-Stages, most cells lining the CC are multiciliated with an advance maturation and differentiation state and only few cells are uniciliated [48]. In R-stages, but not in the NR-stages, SCI induces a massive and transient proliferation of Sox2/3^+^ progenitor that is required for proper spinal cord regeneration, and formation of new neurons [46, 47]. In R-stages, glial cells closely associated with growing axons in the ablation gap, suggesting a possible role for them in generating a glial bridge to aid in axonal regeneration [42]. Little evidence of glial scar formation in non-regenerative stages of *Xenopus* has been reported, so far, scar tissue was found encapsulating the end of the spinal cord lesion in post-metamorphic frogs [44].

Here, we compare the cellular response to SCI of the SC central canal, between the R- and NR-stages of *Xenopus laevis*. By using electron microscopy, we found a very different cellular response between both stages. In R-stage animals, the spinal cord and CC organization were rapidly restored. During regeneration, cells lining the CC seal the injured stumps already at 2 days after injury, activating a proliferative response followed by formation of rosette-like structures in the ablation gap. The CC was filled with cellular material including cells with similar morphology to the cells lining the central canal, neurons, axon, and even synaptic structures. Twenty days after transection, the spinal cord was almost regenerated. A very different response was detected in NR-stage animals, mostly damaged tissue was observed during the first week, with no clear closure of the stumps. The ablation gap was filled with fibroblast-like cells, and deposition of ECM components, and no reconstruction of the spinal cord was observed. These differences in histological response were confirmed by cellular markers analysis. In R-stage animals, a transient increase of Vimentin, Fibronectin and ECM material was detected, contrary to a sustained accumulation of most of these markers, and CSPGs, in the NR-stage.

For a more detailed study of the cellular response, we prepared a transgenic line using the zebrafish *gfap* regulatory regions to drive EGFP expression. Characterization of this transgenic line showed expression in radial glial cells in R-stages, and astrocytes in NR-stage froglets. RNAseq analysis of the cells expressing the transgene in R-stage, demonstrated that they correspond to NSPCs. At the R-stage spinal cord, injury activates proliferation of NSPCs that differentiate into neurons. Ablation of these cells abolishes proper regeneration, confirming that are necessary for a functional regeneration of the spinal cord at NF stage 50.

## Results

### Cellular response to injury in regenerative and non-regenerative stages

The cellular organization of the spinal cord CC in *Xenopus laevis* changes between regenerative and non-regenerative stages [48]. To determine the cellular response to spinal cord injury between regenerative (R-stages, NF stage 50) and non-regenerative (NR-stages, NF stage 66) stages*,* we performed a detailed cellular analysis.

**Fig. 1:**
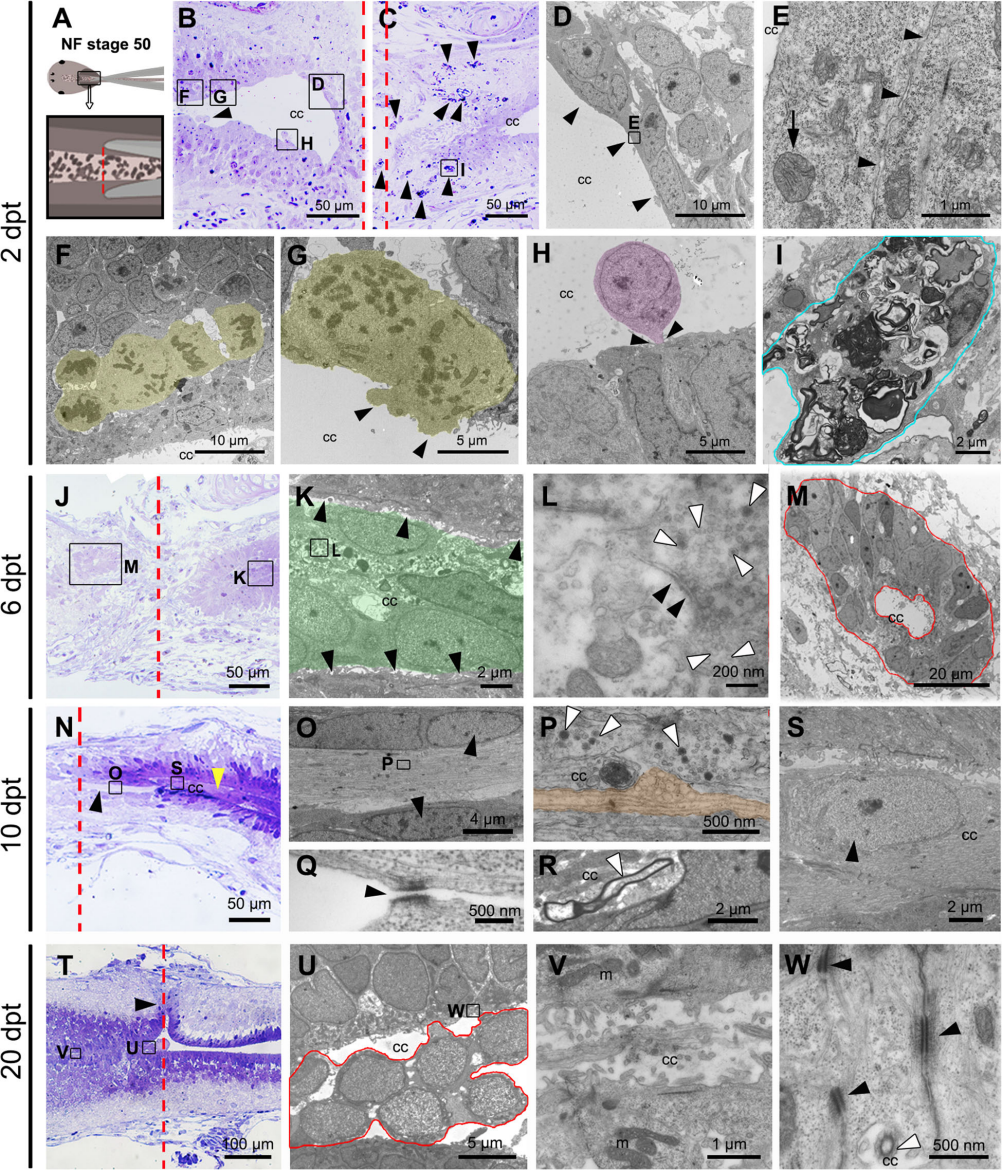
Cellular response to injury in Regenerative Stage. **a** Cartoon of spinal cord injury in NF stage 50. **b**, **c** Semithin sections of the **b** rostral and **c** caudal stumps at 2 days post transection (dpt) stained with methylene blue. Arrowheads in panel C showed macrophages. **d-i**; **k-m**; **o-s**; **u**, **v** Correspond to ultrathin sections observed by transmission electron microscopy. **d-i** Different regions of the spinal cord at 2 dpt; **d** cells lining the central canal (cc) closing the rostral stump (black arrowhead); **e** mitochondrial swelling (black arrow) observed in cells from panel D; black arrowheads depict the separation between two cells; **f, g** mitotic clusters of cells (yellow shadow); **h** cell undergoing extrusion (purple shadow); **i** macrophage from panel C from the injured site. **j** Semithin section at 6 dpt. **k-m** Different regions of the spinal cord at 6 dpt; **k** cells in the central canal of the caudal stump (green shadow), without contact with ependymal cells (black arrowheads); **l** synaptic density (black arrowheads) and synaptic vesicles (white arrowheads); **m** cells forming a rosette structure in the ablation gap (red shadow). **n** Semithin section at 10 dpt. **o-s** Different regions of the spinal cord at 10 dpt; **o** a bundle of unmyelinated axons surrounded by ependymal cells (black arrowheads); **p** unmyelinated axon (orange shadow), and synaptic vesicles (white arrowheads); **q** desmosome junction (black arrowhead) between ependymal cells next to unmyelinated axons; **r** a myelinated axon (white arrowhead) in the cc of the caudal stump; **s** neuronal nuclei in the cc of the caudal stump (black arrowhead). **t** Semithin section at 20 dpt. **u-w** Different regions of the spinal cord at 20 dpt; **u** cells in the cc (red line); **v** ependymal cells with regular shape in the regenerated spinal cord with apical mitochondria (m); **w** desmosome junctions between the regenerated ependymal cells (black arrowheads). The red dotted lines indicate the injured site (**a**, **b**, **c**, **j**, **n**, **t**)

The spinal cord of R-stage animals was injured by full transection as described previously [40] (Fig. 1a), and tissues were analyzed by light and electron microscopy at different days post transection (dpt). At 2 dpt (Fig. 1b, d), a complete sealing of the rostral stump was observed (Fig. 1b, arrowheads in Fig. 1d). The cells lining the CC close to the injury site were not affected by the lesion. To identify ultrastructural changes in CC cells after SCI, we analyzed ultrathin sections. Cells lining the CC, characterized in the control as type I, II or III [48], lack junction complexes compared to controls (Fig. 1e, arrowheads), contain swelled mitochondria in their apical pole (Fig. 1e, arrow), and frequent centriolar satellites were found (see supplementary material, Fig. S1A, arrowheads). As expected, we identified abundant cells showing mitotic figures (27,75 mitotic cells/μm^2^ × 10^5^, sd. 4,32) indicating cell division [46, 47]. Almost half of the cellular clusters undergoing cell division have no contact (12 mitotic cells/μm^2^ × 10^5^, sd. 2,55) with the lumen of the central canal (Fig. 1f), while the other half (15,75 mitotic cells/μm^2^ × 10^5^, sd. 2,59) are in direct contact with it (Fig. 1g). Although in a lower proportion, cell division in the CC has been also observed in uninjured animals [47, 48]. Conspicuous among the cells lining the CC was the presence of donut- and phone-like shaped mitochondria, phenome not observed in control animals (see supplementary material, Fig. S1B, arrowheads).

Interestingly, we have found cells in the CC that were being extruded toward the lumen (Fig. 1b, h arrowheads). In vivo time-lapse imaging of zGFAP::EGFP transgenic animals (more details in the following sections) reveals that at 2 dpt EGFP^+^ and EGFP^−^ processes and cell bodies from the cellular layer lining the central canal are being extruded into the central canal (see supplementary material, Fig. S2). Regarding infiltration in the injury site we found blood cells (19,75 red blood cells/μm^2^ × 10^5^, sd. 3,49), macrophages (17,25 macrophages/μm^2^ × 10^5^, sd. 1,29) (Fig. 1c, arrowheads) which were phagocytosing cellular debris (Fig. 1i) in the lesion site at 2 dpt (see supplementary Fig. S1G, I). On the contrary, very few neutrophils were detected (see supplementary material, Fig. S1C).

At 6 dpt cells accumulate in the lumen of the central canal (Fig. 1j, k). These cells showed a higher nucleus/cytoplasm ratio compared to the cells lining the CC, have a lax chromatin, a scarce cytoplasm with few organelles, and the absence of cellular junctions between them or with cells of the spinal cord (Fig. 1k, arrows). Interestingly, some cellular expansions within the lumen exhibited a high density of light vesicles, and few small dense core vesicles (Fig. 1l, white arrowheads) in near proximity to structures reminiscent of postsynaptic densities (Fig. 1l, black arrowheads). At this time, clusters of 20–24 cells forming rosette like structures were found in the ablation gap (Fig. 1j, m). These cells are very similar to those found in the uninjured central canal; resembling type III cells previously described [48]. These cells have a characteristic neuroepithelium organization; with a basal lamina containing Collagen (see supplementary material, Fig. S1D), a high nucleus/cytoplasm ratio, apical mitochondria, abundant apical interdigitations with adherent cell junctions (see supplementary material, Fig. S1E), a high content of intermediate filaments (see supplementary material, Fig. S1F), and the presence of a cilium (data not shown). Blood cell infiltration was considerable reduced at 6 dpt compared with 2 dpt in R-stage, we found 0,5 red blood cells/μm^2^ × 10^5^ (sd. 0,5) and 8,5 macrophages/μm^2^ × 10^5^ (sd. 2,69) in the lesion site at 6 dpt (see supplementary Fig. S1G, and I).

At 10 dpt some continuity between the rostral and caudal stumps was observed (Fig. 1n). Surprisingly, abundant bundles of axons were found inside the lumen of the caudal central canal (Fig. 1n, o). These bundles were mainly composed by unmyelinated axons (Fig. 1p, orange) that were in close contact with synaptic vesicles (Fig. 1p, arrowheads), but also some myelinated axons were detected (Fig. 1r, arrowhead). The axon bundles were surrounded by cells with a fusiform nucleus that have a lax chromatin (Fig. 1o, arrowheads), and desmosome-like junctions were found between those cells (Fig. 1q). These cells in close contact with axons have a condensed chromatin resembling a neuronal morphology (Fig. 1s). In line with the observation at 6 dpt, rosettes and some cells are still present in the ablation gap (data not shown), and the central canal, respectively.

Finally, as described before [47] at 20 dpt we observed an almost complete reconstruction of the spinal cord (Fig. 1t). However, the continuity of the central canal was not perfect (Fig. 1t, arrowhead). Importantly, the cells lining the central canal recover its normal organization as a pseudo-stratified epithelium (Fig. 1u), with apical mitochondria, microvilli (Fig. 1v), and desmosome cell junctions (Fig. 1w). Few cells remained inside the lumen of the central canal (Fig. 1u, red line).

**Fig. 2:**
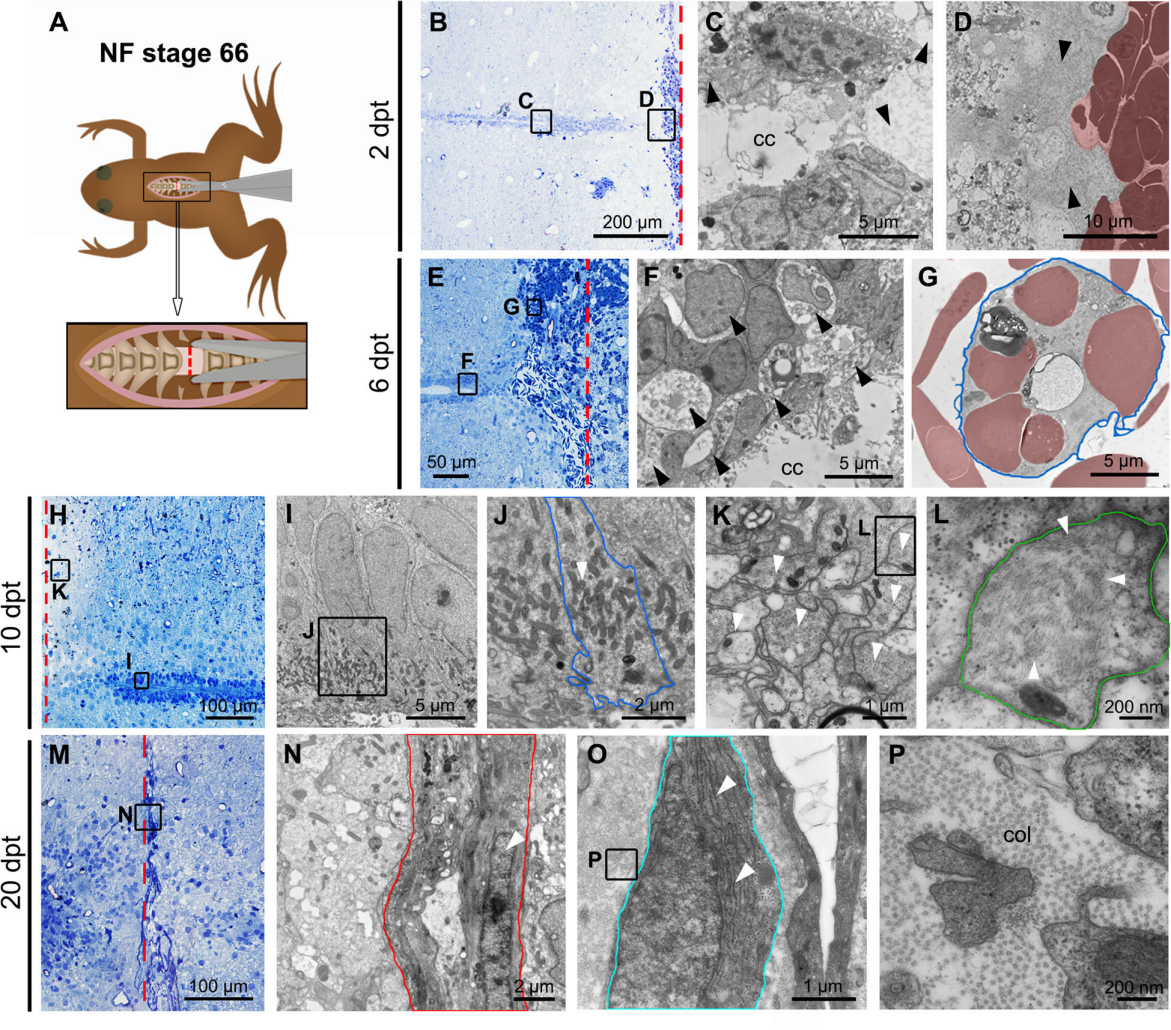
Cellular response to injury in Non-Regenerative Stage. **a** Cartoon depicting the process of spinal cord injury in NF stage 66. **b** Semithin section at 2 dpt. **c**, **d**; **f-g**; **i-l**; **n-p** Correspond to ultrathin sections observed by transmission electron microscopy. **c, d** Different regions of the spinal cord at 2 dpt; **c** central canal (cc) next to the injured site (black arrowheads); **d** extracellular matrix and red blood cells (red shadow) in the injury site. **e** Semithin section at 6 dpt. **f, g** Different regions of the spinal cord at 6 dpt; **f** ependymal cells in the rostral stump (black arrowheads); **g** macrophage (blue line) engulfing red blood cells (red shadow) in the cc. **h** Semithin section of the caudal stump at 10 dpt. **i-l** Different regions of the spinal cord at 10 dpt; **i** ependymal cells near to the injured site; **j** mitochondria (white arrowhead) in the apical surface of ependymal cell (blue line) in contact with the cc; **k** glial cell processes next to the injured site (white arrowheads); **l** intermediate filaments (white arrowheads) in the glial process (green line). **m** Semithin section at 20 dpt. **n-p** Different regions of the spinal cord at 20 dpt; **n** ablation gap (red lines) filled with fibroblast-like cell (white arrowhead), and surrounded by extracellular matrix; **o** microglial-like cell (cyan line) with abundant rough endoplasmic reticulum (white arrowheads); **p** abundant Collagen (col) fibers (dots) in the injured site. The red dotted lines indicate the injured site (**a**, **b**, **e**, **h**, **m**)

A similar analysis was carried out in NR-stage animals. For this the spinal cord of animals at stage 66 was transected as described [40] (Fig. 2a), and the CC was analyzed at different times after injury. Contrary to the response observed in the R-stage, at 2 dpt the cells lining the CC were severely damaged, with loss of their intracellular components similar to necrotic cells, and the empty spaces between cells leads to a strong disorganization and loss of the pseudo-stratified epithelial organization (Fig. 2b, c, arrowheads). An important loss of intracellular organelles is observed in the remaining ependymal cells (data not shown). In addition, we observed a massive invasion of the ablation gap with blood cells (316,25 red blood cells/μm^2^ × 10^5^, sd. 23,28 and 1,25 macrophages/μm^2^ × 10^5^, sd. 0,83) (Fig. 2b, d; red shadow; supplementary Fig. S1H, J), and the deposition of extracellular matrix (ECM) components (Fig. 2d, arrowheads). A massive disorganization of the central canal, is still observed at 6 dpt, together with a sustained increase in the extracellular spaces between cells, and the presence of vacuolated cells (Fig. 2e, f; see arrowheads). In addition, a massive presence of blood cells (490,5 red blood cells/μm^2^ × 10^5^, sd. 42,5; and 137,25 macrophages/μm^2^ × 10^5^, sd. 20,87) (Fig. 2e, g; supplementary Fig. S1H, J), and very few microglia was still detected in the injury site.

At 10 dpt, the CC cells have recovered some epithelial organization (Fig. 2h, i), and have an abundant number of mitochondria in the apical surface (Fig. 2i, j) which resemble the lateral ependymal cells described before [48]. Unlike the response at the R-stage, froglets at the NR-stage are characterized by the absence of proliferation and rosette-like structures. Ten days after injury the borders of the rostral and caudal stump were surrounded by glial processes (Fig. 2k, white arrowheads), containing abundant intermediate filaments (Fig. 2l, white arrowheads). Finally, at 20 dpt the presence of red blood cells, and immune cells in the injury site had decreased (Fig. 2m), and the ablation gap is mainly filled by fibroblast-like cells, characterized by long nuclei (Fig. 2n, white arrowhead), a very dilated rough endoplasmic reticulum (Fig. 2o, white arrowheads), and an ECM containing abundant Collagen fibers (Fig. 2p). These morphological features correlate with the complete lack of swimming capacities at 20 dpt in NR-stage [46, 47].

In summary, R-stages revealed a dynamic regenerative process characterized by a fast response of cells lining the central canal to rapidly seal the injured stumps, activating a proliferative response followed by formation of rosette-like structures in the ablation gap. In addition, cells are extruded into the lumen of the central canal, which is also filled with axons and synaptic vesicles. Regenerated spinal cords, although not always with a perfect morphology, indicates an efficient resolution of the regeneration at 20 dpt. On the contrary, in the NR-stage, cells lining the central canal are deeply affected after injury, and instead, red blood cells and macrophages populate the injury site. After several days, cells lining the canal recover their ultrastructure, but the injury site is filled with glial cells processes, fibroblast and collagen fibers, reminiscent of the glial scar described in mammals, and a proper recovery of the spinal cord was not observed. A higher number of red blood cells and macrophages were observed in NR-stage at 2 and 6 dpt. Remarkable, although red blood cells and macrophages were observed after injury in R-stage at 2 dpt, a significant reduction of blood cells was observed at 6 dpt. These results showed that in R-stage the red blood cells and macrophages infiltration response is earlier and resolved faster than in NR-stage.

### Analysis of the presence of glial scar markers in response to spinal cord injury

One of the hallmarks of the cellular response to SCI in mammals is the formation of a glial scar composed of a fibrotic scar, and an astroglial scar border [6]. This scar is composed of different cell types including astrocytes, microglia, pericytes, and inflammatory and meningeal cells, together with ECM components such as Fibronectin, CSPGs, and Collagen among others [17, 18]. To evaluate the formation of a glial scar in R- and NR-stages in response to injury we evaluated the presence of some of these markers at different days after injury.

**Fig. 3:**
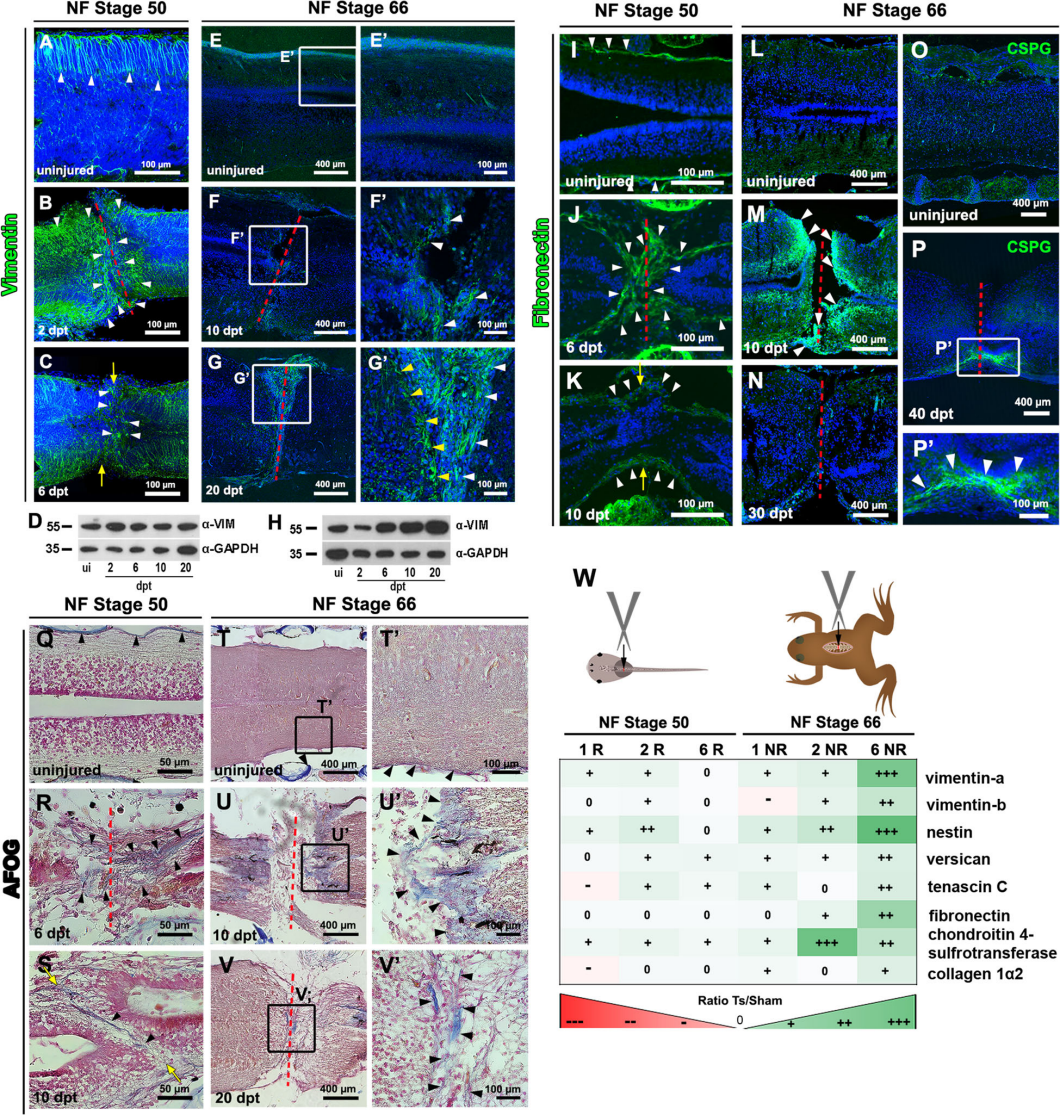
Glial cell and extracellular matrix response to spinal cord injury in R-Stage and NR-Stage. **a-c** Immunostaining against vimentin in **a** uninjured, and at **b** 2 and **c** 6 dpt from animals at NF stage 50. **d** Western blot against Vimentin (Vim) and GAPDH of spinal cords samples obtained from uninjured (ui), and at 2, 6, 10 and 20 dpt in animals at NF stage 50. **e-g** Immunostaining against Vimentin in uninjured **(e-e’),** and at **(f, f’)** 10 and **(g-g’)** 20 dpt from animals at NF stage 66. **h** Western blot against Vim and GAPDH of spinal cords samples obtained from uninjured (ui), and at 2, 6, 10 and 20 dpt in animals at NF stage 66. **i-o** Immunofluorescence against fibronectin in **i** uninjured, **j** 6 dpt, and **k** 10 dpt in NF stage 50; and **l** uninjured, **m** 10 dpt, and **n** 30 dpt in animals at NF stage 66. **o-p** Immunofluorescence against CSPG in **o** uninjured, and **p-p’** at 40 dpt in NF stage 66. **q-v** AFOG staining shown Collagen (blue), cells (orange) and Fibrin (red) in **q** uninjured, and at **r** 6 and **s** 10 dpt in animals at NF stage 50, and in **t-t’** uninjured, and at **u-u’** 10 and **v-v’** 20 dpt from animals at NF stage 66. **w** Analysis of gene expression change upon spinal cord injury comparing injured animals (Ts) with control sham (sham) surgery at 1, 2 and 6 days after injury in NF stage 50 (1R, 2R and 6R), and NF stage 66 (1NR, 2 NR and 6 NR). Colored and crosses scale indicates the level of increase upon injury in green (+, ++, +++) and decrease in red (−, −−, −−−), data obtained from a previous RNAseq analysis [49]. The red dotted lines (**b**, **f**, **g**, **j**, **m**, **n**, **p**, **r**, **u**, **v**) and yellow arrows (**c**, **k**, **s**) indicate the injured site. Nuclei stained with Hoechst in blue (**a-c**; **e**-**g**; **i**-**p**)

First, we studied the expression of Vimentin, an intermediate filament that is a marker of glial cells. In R-stage, Vimentin was found in radial filaments located at the dorsal domain in the uninjured spinal cord (Fig. 3a, white arrowheads), as previously described [49]. Two days after injury, there was an increase in the number of cells expressing Vimentin especially in the ablation gap and the regions close to the injury site (Fig. 3b, white arrowheads). Although a decrease in cells expressing Vimentin was observed at 6 dpt, they are still higher than those detected in uninjured animals (Fig. 3c). For a more quantitative analysis, the region of the spinal cord surrounding the injury site was isolated and homogenized for western blot and quantification analysis. In this analysis, no change of Vimentin levels was observed at 2, 6, 10 and 20 dpt in R-stage (Fig. 3d, see supplementary material, Fig. S3A, B, E). In NR-stage froglets, Vimentin was only expressed in blood vessels in uninjured froglets (Fig. 3e-e’). At 10 dpt, Vimentin was detected in cells with radial processes at the edges of the lesion (Fig. 3f-f’, withe arrowheads) and later, at 20 dpt, many of the Vimentin positive cells with radial processes were now present in the ablation gap, most probably representing glial cells forming a glial scar (Fig. 3g-g’). The observed rise in Vimentin correlates with the increase in radial processes previously detected by EM analysis, in which we observed processes with intermediate filaments at the edge of the lesion (Fig. 2k, l). This increase in the levels of Vimentin at later days (10 and 20 dpt) was confirmed by western blot quantification analysis (Fig. 3h, see supplementary material, Fig. S3B).

The presence of the ECM components typical of the glial scar in mammals was evaluated. Fibronectin is expressed in the meninges in uninjured conditions in R-stage (Fig. 3i). However, at 6 dpt a clear increase in Fibronectin was detected in cells that seal the rostral and caudal stump, most probably corresponding to meningeal cells, and was also found in more cells in the injury site (Fig. 3j). This increase is transient, and the levels of Fibronectin return to almost normal at 10 dpt, being only expressed in the meningeal layer of the spinal cord (Fig. 3k). Fibronectin was almost not detected in froglets (Fig. 3l), and a similar response but more delayed, was observed. At 10 dpt, there is an increase in fibronectin deposition in the lesion site in the rostral and caudal stumps (Fig. 3M), and the levels are normal around 30 dpt (Fig. 3n). Similarly, we performed analysis of CSPGs, in NR-stages. Uninjured froglets showed expression of CSPGs only in blood vessels and vertebrae (Fig. 3o), and after injury a clear increase in the lesion site was observed, and was still present at 40 dpt (Fig. 3p-p’). CSPGs were not detected in the spinal cord before or after injury in R-stage animals (data not shown). For further analysis of Collagen deposition, spinal cord sections were stained with Acid Fuchsin Orange G (AFOG), which labels Collagen in blue, cells in orange and Fibrin in red. Collagen was expressed in the meningeal layer in uninjured R-stage and froglets (see arrowheads, Fig. 3q and t-t’). However, at 6 dpt the levels of Collagen increased in the lesion site in R-stage (Fig. 3r), and at 10 dpt in froglets (Fig. 3u-u’). Interestingly, in R-stages the levels of Collagen decreased, at 10 dpt (Fig. 3S), but high levels of Collagen were still present in the lesion site in froglets at 20 dpt (Fig. 3v-v’). The increase of Collagen in R-stage at 6 dpt and in NR-stage at 10 and 20 dpt were confirmed by quantification (see Supplementary Fig. S3G).

For an unbiased comparison of the expression of glial markers in response to injury in R- and NR-stages we explored a data set from a high-throughput transcriptome analysis performed previously [49]. We studied the levels of expression of 8 transcripts including: the intermediate filaments *vimentin* (aloalleles a and b) and *nestin*, the ECM components *versican*, *tenascin-c*, *fibronectin*, *collagen type 1 alpha 2*, and the enzyme *chondroitin 4-sulfotransferase*, that is important for the synthesis of CSPGs. Of note, we found that SCI increased significantly the levels of these transcripts at 6 dpt in NR-stage froglets, but not in R-stage animals (Fig. 3w), providing further support to the different glial response in both stages.

In summary, we observed a scar formation in response to injury in NR-stage, which is absent in R-stage. A clear difference in the expression of glial scar makers in response to injury was found in R- and NR-stages. On the one hand, we found a transient increase of Vimentin, Fibronectin and Collagen proteins in R-stages, and no important changes at the RNA levels of glia scar markers. On the contrary, in froglets, a delayed and sustained increase in the protein levels of Vimentin, Collagen, and CSPGs was observed, together with a steady increase of their mRNAs levels.

### Characterization of the zGFAP::EGFP transgenic line

For a better understanding of the cellular response triggered by SCI in R- and NR-stages, we decided to prepare a transgenic line that could label most of the cells in the central canal. Although it has been demonstrated that a GFAP gene was lost in *Xenopus* during anuran evolution [50], we decided to use the zebrafish GFAP (zGFAP) regulatory promoter regions to prepare a transgenic line in *Xenopus* because of the following reasons: i) based on evolutionary conservation, it is very possible that the main regulators of the gene-regulatory networks operating in glial cells are maintained, because of that we hypothesized that the regulatory promoter regions of the zebrafish GFAP gene could drive expression of a transgene in the same cells in which it is active in zebrafish; ii) a transgenic line using a 11.6 kb region of zGFAP regulatory sequences had been reported and showed proper expression of the transgene in glial and neural progenitor cells in zebrafish [51], and iii) GFAP is usually expressed in many of the cells that are present in the CC including among others radial glial cells, neural stem, neural progenitors, astrocytes and ependymal cells [51].

Before the generation of the transgenic line, and to test our assumption that the zGFAP promoter will drive proper expression in *X. laevis* spinal cord, we electroporated the spinal cord with a construct in which EGFP expression is driven by the zGFAP regulatory sequences (zGFAP::EGFP), or a control transgene driven the expression of EGFP under a constitutive active promoter (CAG::EGFP). Electroporation of CAG::EGFP revealed an abundant and ubiquitous expression in most of the cells of the spinal cord (see supplementary material, Fig. S4a-c); compared to a more specific and selective expression after electroporation of the zGFAP::EGFP construct, which labeled a specific group of cells in the spinal cord that have a radial glial cell morphology (see supplementary material, Fig. S4d-f). This analysis suggests that the zebrafish regulatory regions drive proper expression in *Xenopus*. Because of this, we prepared a transgenic line in *X. laevis* using the same genomic region from the zGFAP described before [51]. The line obtained was named Xla.Tg(Dre.gfap:EGFP)^Larra^, for short zGFAP::EGFP.

**Fig. 4:**
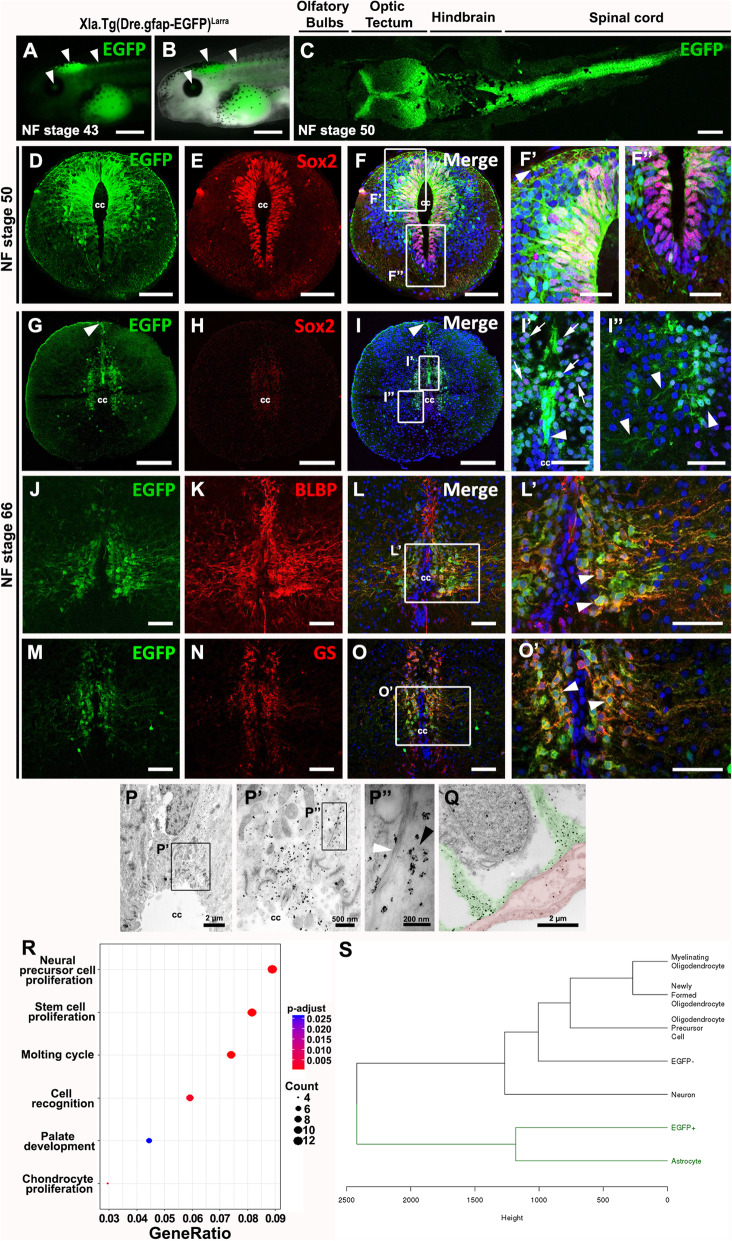
Zebrafish regulatory regions of GFAP drive expression of EGFP in neural stem and progenitor cells, and astrocytes in *Xenopus laevis* spinal cord. **A-C** Lateral view of EGFP expression in the central nervous system at **A**, **B** NF-Stage 43, and **C** NF-Stage 50. **A** EGFP expression in the eye, brain and spinal cord (arrowheads). **B** EGFP/brightfield merge. **C** Dorsal view of EGFP expression in the optic tectum, hindbrain and spinal cord at NF stage 50. **D-F** Double staining against **D** EGFP and **E** Sox2. Panels **F** showed merge image, and **F’, F”** magnifications of the dorsal and ventral cells surrounding the central canal. **G-O’** Characterization of EGFP cells by double staining at NF stage 66. **G-I”** EGFP and Sox2; **J-L’** EGFP and Brain lipid-binding protein (BLBP); and **M-O’** EGFP and Glutamine synthase (GS). Nuclei are label in blue with Hoechst. **P-Q** Immunogold staining against EGFP at NF stage 50. **P** EGFP^+^ cell in contact with the central canal. **P’** Magnification of square in P. Expression of EGFP is visualized by the black dots of the gold staining. **P”** Magnification of square in P’. Gold staining (black arrowhead) in close apposition with filaments (white arrowhead). **Q** Endfeet from an EGFP^+^ cell (colored green) in close contact with blood vessel (colored red). **R** Gene ontology analysis of the RNAseq from EGFP^+^ cells revealed the stem cell/neural precursor cell identity of these cells. **S** Dendrogram of EGFP^+^ cells and EGFP^−^ cells showing the hierarchical clustering of EGFP^+^ cells with astrocytes and EGFP^−^ cells with neurons and oligodendrocytes. Scale bar: C, F’-F″: 20 μm; A-B, D-F, I’-I″, L’, O’: 50 μm; G-I, J-L, M-O: 200 μm

EGFP expression in the transgenic line was detected in the central nervous system (CNS) throughout development (see supplementary material, Fig. S4g-j’). At NF stage 43 and 50 the transgene was expressed in the retina, tectum, cerebellum and spinal cord, but not in the more anterior region of the CNS (Fig. 4a-c). Coronal sections of the cervical, thoracic and lumbar spinal cord showed expression of EGFP in cells, mainly in the dorsal portion of the spinal cord, that have a radial glia morphology, with their apical surface lining the central canal and a long projection to the meningeal layer (Fig. 4d and see supplementary material, Fig. S4K). Most EGFP^+^ cells also expressed Sox2, a marker of neural stem progenitor cells (NSPCs), but not all Sox2^+^ cells expressed EGFP (Fig. 4e-f” and see supplementary material, Fig. S4L-M”). We noted that the region of the spinal cord with cells expressing EGFP corresponds to the same region containing cells that are actively proliferating in uninjured animals, as demonstrated before by the incorporation of thymidine analogues [47, 48, 52].

EGFP^+^ cells were also found in the spinal cord in NF stage 66 froglets, but have a very different shape and distribution. At this stage only a reduced group of EGFP^+^ cells are in contact with the CC, mainly on the most dorsal portion (Fig. 4g and i), and extend a dense array of projections towards the meningeal layer (Fig. 4g, i and i’). The most abundant cells expressing EGFP correspond to cells that are not in contact with the CC, but also have cellular projections (Fig. 4g, i, i” arrowheads). As shown previously, low levels of Sox2 expression were detected in cells lining the central canal, particularly in the subependymal layer co-localizing with EGFP (Fig. 4h, i”). Most EGFP^+^ cells at NF stage 66 co-expressed the Brain lipid binding protein (BLBP) (Fig. 4j-l’), and Glutamine synthase (GS) (Fig. 4m-o’), two markers of radial glial cells during early development, and markers of astrocytes at later stages. Base on their morphology, location, and co-expression of other markers we propose that at NF stage 66 most EGFP^+^ cells at the subependymal layer correspond to astrocytes.

For a more accurate identification and morphological characterization of the cells expressing EGFP in R-stage, we carried out immunogold staining using anti-EGFP antibodies. Gold particles were found in the cytoplasm of cells that are in contact with the central canal (Fig. 4p) and contain intermediate filaments (Fig. 4p’, p”). Gold particles were also found on cellular projections that were in direct contact with blood vessels on the meningeal side of the spinal cord (Fig. 4q). Based on their morphology, co-expression of Sox2 and proliferative activity we envision that most EGFP^+^ cells in the spinal cord of R-stage animals correspond to NSPCs with a radial glial morphology. According to our previous characterization of the cells lining the central canal, EGFP^+^ cells in the R-stage correspond to cells type II and III and is almost not detected in froglets [48].

To unequivocally address the identity of EGFP^+^ cells in the zGFAP::EGFP transgenic animals at R-stage (NF stage 50) we separated EGFP^+^ and EGFP^−^ cells using fluorescent activated cell sorter (FACS), and performed RNAseq in a single biological replicate for each cell population. Total RNA from EGFP^+^ and EGFP^−^ cells was sequenced, and the reads mapped. Bioinformatics analysis (see supplementary material Fig. S5A) identified 1718 transcripts with different levels of expression between EGFP^+^ and EGFP^−^ cells, 147 of them enriched in EGFP^+^ cells, including EGFP with the highest fold of change, and 1571 that showed lower levels of expression in EGFP^+^ cells (see supplementary material, Fig. S5B). Importantly, gene ontology analysis of the genes enriched in EGFP^+^ cells showed that the two main categories of biological processes enriched correspond to neural precursor cell identity and stem cell proliferation categories (Fig. 4r). Furthermore, a cluster dendrogram analysis comparing the gene profile of EGFP^+^ and EGFP^−^ cells with a database of different cells types of the CNS [53] revealed a close correlation between EGFP^+^ cells and astrocytes, that probably include a radial glial cell signature (Fig. 4s). This molecular analysis confirmed that most of the EGFP^+^ cells in the zGFAP::EGFP transgenic line correspond to NSPCs at NF stage 50, and validated these transgenic animals as a bona fide tool to study the response of NSPCs to SCI.

In summary, the use of the zebrafish regulatory regions of GFAP allowed the generation of a *X. laevis* transgenic line in which EGFP expression recapitulates the expected pattern in the CNS. In R-stages, it is expressed mainly in NSPCs with a radial morphology, and later in NR-stages is found primarily in astrocytes, providing a useful and reliable tool to study and characterize the function of these cells in different developmental and regenerative contexts.

### Response of neural stem progenitor cells to spinal cord injury

**Fig. 5:**
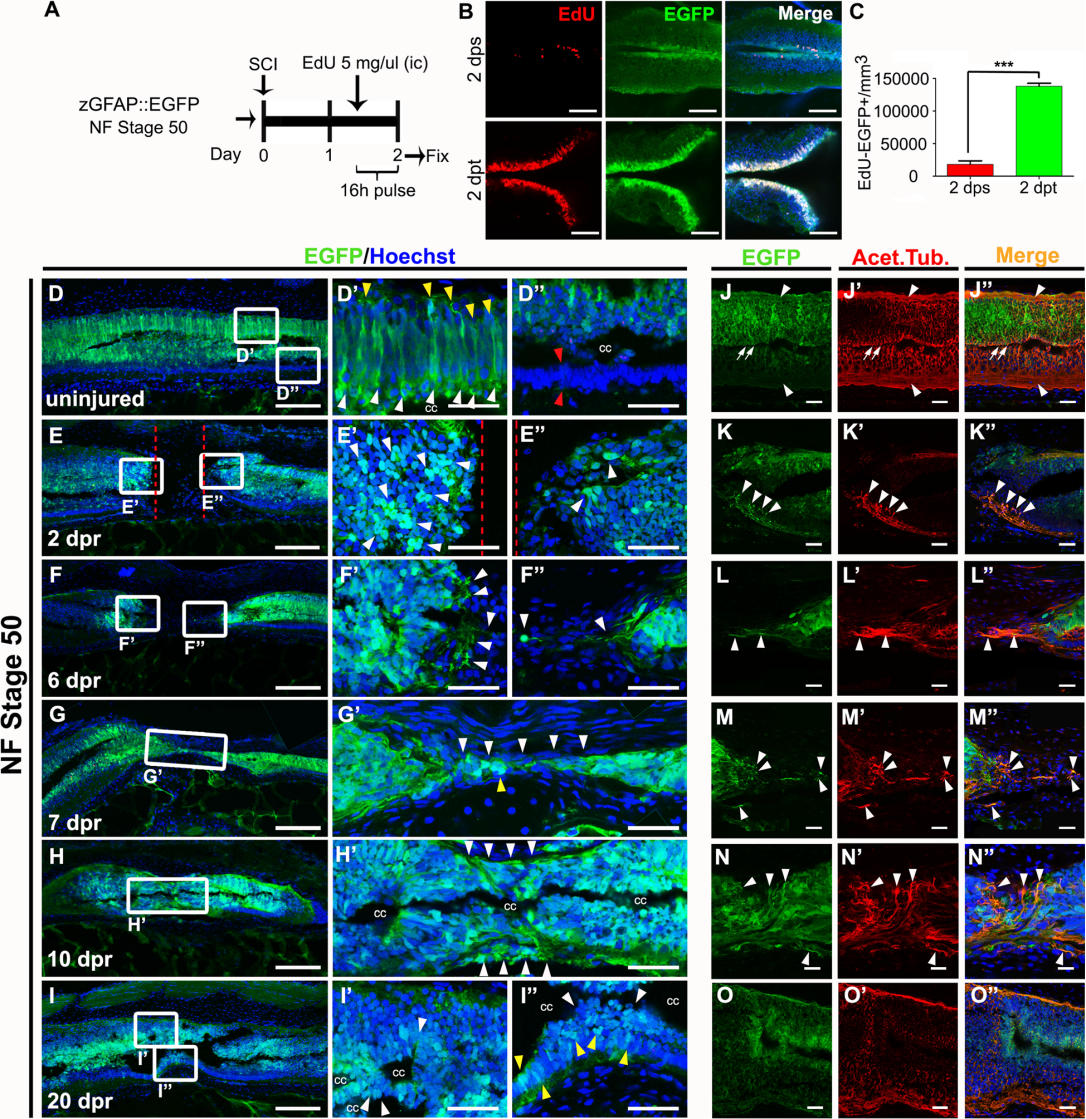
Response to injury of Neural Stem and Progenitor Cells. **a** Scheme of EdU treatment. **b** Click-iT staining for EdU (red), and immunofluorescence against EGFP (green), merge with nuclei (blue) in sham control animals at 2 days post sham operation (dps), and 2 dpt. **c** Graph of EdU-EGFP positive cells per mm^3^ at 2 dps (red bar) and 2 dpt (green bar). t-Test, ***: *p* < 0.001. **d, e, f, g, h, i** Immunofluorescence against EGFP (green) at NF stage 50 in **d** uninjured, **e** 2 days post resection (dpr), **f** 6 dpr, **g** 7 dpr, **h** 10 dpr, and **i** 20 dpr. Magnifications are shown in panels **d’-d”**, **e’-e”**, **f’-f”**, **g’, h’** and **i’-i”**. **j, k, l, m, n, o.** Serial sections from the same preparation shown in panels **d, e, f, g, h, i** double stained for EGFP (green) with the neuronal marker Acetylated tubulin (red), and merge (orange). Nuclei are label in blue with Hoechst. White arrowhead highlights colocalization. Scale bar: **d**, **e**, **f**, **g**, **h**, **i**: 200 μm; **d’-d”**, **e’-e”**, **f’-f”**, **g’**, **h’**, **i’-i”**, **j-j”**, **k-k”**, **l-l”**, **m-m”**, **n-n”**, **o-o**”: 50 μm

We used the transgenic line described above to study the response, and function of NSPCs after SCI in R-stage animals. To evaluate the proliferative response of these cells, transected and sham controls animals were incubated with a pulse of 5-Ethynyl-2′-deoxyuridine (EdU) between 20 and 36 h after injury (Fig. 5a). Low levels of EdU incorporation in EGFP^+^ cells were observed in sham operated animals (Fig. 5b, c). Contrary to that, a massive proliferation of EGFP^+^ cells was observed after SCI (Fig. 5b, c). As a control, EdU^+^ cells were counted in the intestine, an organ with a high proliferation rate, and no change on the proliferative rate was observed (see supplementary material Fig. S6A-C), indicating that the activation of proliferation raised by SCI was specific for NSPCs in the spinal cord. These results are very similar to those reported for the activation of Sox2^+^ cells in R-stage [47] giving further support to the finding that most EGFP^+^ cells at this stage co-express Sox2.

To study in detail the response of EGFP^+^ cells, we fixed animals at different days, and performed immunofluorescence against EGFP in longitudinal sections. Although EGFP perdurance is not a bona fide method to study cell fate, because it could also include new cells expressing EGFP, it could provide an idea of the fate of existing or new EGFP^+^ cells. To allow a more detailed analysis of the cellular responses in the ablation gap, we performed a resection of the spinal cord that implicates the complete removal of a piece of the spinal cord of approximately 200 μm. In uninjured animals, but now in a longitudinal section, the EGFP^+^ cells showed their radial morphology and its dorsal and lateral location (Fig. 5d-d”). At 2 days post resection (dpr), both ends of the spinal cord were approximately 200 μm apart (Fig. 5e), and many round shaped EGFP^+^ cells were found at the edges of the rostral and caudal stumps (Fig. 5e’, e”, arrowheads). Interestingly, at 6 dpr, EGFP^+^ cellular processes started to extend from the rostral and caudal stumps towards the ablation gap (Fig. 5f-f”, arrowheads). At 7 dpr some of these processes were even able to cross the complete ablation gap (Fig. 5g, g’, arrowheads). At 10 dpr, EGFP^+^ cells populated the injured site (Fig. 5h, h’) and some reorganization of the central canal is observed (Fig. 5h’). At 20 dpr, some recovery of the anatomy of the spinal cord was observed (Fig. 1t, and [46, 47]). EGFP^+^ cells were starting to acquire their normal location; however, a radial glial morphology was not observed, and these cells were now present in the ventral level (Fig. 5i-i”).

Regarding the cellular processes extending into the ablation gap observed at 6–7 dpr we hypothesized two alternatives: i) they could correspond to glial extensions that can provide a substrate for axon growth and pathfinding, something that has been proposed before [42] or ii) in agreement with our previous findings on the role of neurogenesis on spinal cord regeneration [47], these processes could be axons from the new neurons generated from the EGFP^+^ cells, that because of the half-life of the EGFP protein allowed the study of the cell fate of the EGFP^+^ cells. To analyze these two possibilities, we performed immunofluorescence against Acetylated tubulin, which labels axons and cilia, in the same time points depicted above. As expected, in uninjured animals, Acetylated tubulin does not co-localized with EGFP in axons (Fig. 5j-j”, see arrowheads), but there is co-localization in cells in the central canal probably because Acetylated tubulin is present in ciliated cells (Fig. 5j-j”, see arrows). However, at 2 days after injury a clear co-localization of Acetylated tubulin with EGFP was detected, primarily at the edge of the stumps in structures that are reminiscent of axons and axon growth cones (Fig. 5k-k”, arrowheads). Importantly, at 6 and 7 dpr most of the EGFP^+^ cellular processes extending into the ablation gap co-localized with Acetylated tubulin (Fig. 5l-l”, m-m”). Something similar was observed at 10 dpr (Fig. 5n-n”). An expression pattern of Acetylated tubulin like the uninjured spinal cord is observed at 20 dpr, however, some co-localization of Acetylated tubulin and EGFP was still observed in the axonal tracts (Fig. 5o-o”, see arrowheads).

These results support the hypothesis that the EGFP^+^ cellular processes crossing the ablation gap correspond to axons because of their morphology, and the co-expression of acetylated tubulin. The fact that they are EGFP^+^ indicates that most probably they correspond to new neurons formed from the NSPCs present in the central canal.

### Differentiation of neural stem progenitor cells in spinal cord regeneration

**Fig. 6:**
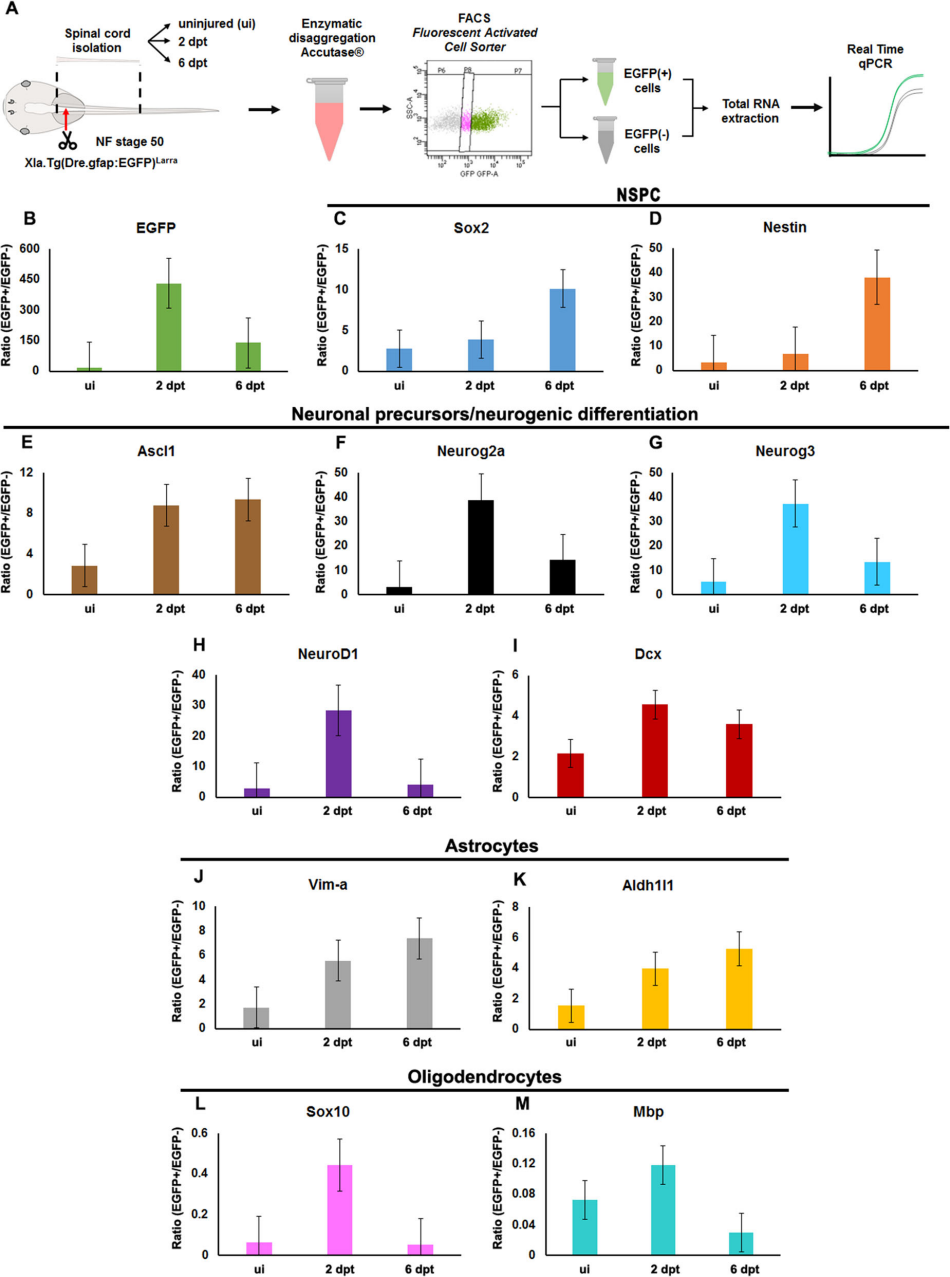
Analysis of the differentiation of Neural Stem Progenitor Cells in response to spinal cord injury. **a** Diagram of the experimental procedure. **b-m** Graphs of the ratio in the mRNA levels for the indicated genes between the EGFP^+^ cells and EGFP^−^ cells in uninjured (ui), 2 and 6 dpt. **b** EGFP, **c, d** NSPCs markers: **c** *sox2*, **d** *nestin*. **e-i** neuronal precursor/neurogenic differentiation markers: **e** *achaete-scute homolog 1* (*ascl1*), **f** *neurogenin2a* (*neurog2a*), **g** *neurogenin3* (*neurog3*), **h** *neurod1*, **i** *doublecortin* (*dcx*). **j, k** Astrocytes markers: **j** *vimentin-a* (*vim-a*), **k** *aldh1l1*. **l**, **m** Oligodendrocytes markers: **l** *sox10*, **m** *myelin basic protein* (*mbp*). *n* = 2–3 samples. Standard error bar is included in each graph

To further evaluate NSPCs, we took advantage of the persistence of EGFP expression. EGFP^+^ and EGFP^−^ cells were isolated by FACS before and after injury, and the expression levels of the following markers were measured by RT-qPCR (Fig. 6a): i) *sox2* and *nestin*, for NSPCs; ii) *neurogenin3*, *achaete-scute homolog 1 (ascl1)*, *neurogenin2a*, *doublecortin (dcx)*, and *neurod1*, for neuronal precursors and neurogenic differentiation; (iii) *aldehyde dehydrogenase1l1 (aldh1l1)* and *vimentin-a*, for astrocytes; and the *myelin binding protein (mbp)*, and *sox10* for oligodendrocytes.

An increase of approximately 450 and 130 times, was observed in the ratio of EGFP levels between EGFP^+^ and EGFP^−^cells, at 2 and 6 dpt, respectively. These ratios were many times higher than the ratios detected in uninjured animals (Fig. 6b), probably explained by the increase on the total number of EGFP^+^/NSPCs, because of its massive proliferation induced by SCI (Fig. 5c, and [47]). Supporting the increase in the proliferation of NSPCs induced by transection, we observed higher ratios of *sox2* and *nestin*, between EGFP^+^ and EGFP^−^ cells, at 2 and 6 dpt (Fig. 6c, d). The most probable explanation for this rise is an increase in the number of EGFP^+^ cells; however, we cannot discard the possibility that the higher ratios were also explained by an increased expression of these genes in each EGFP^+^ cell.

Interestingly, the early neurogenic markers *ascl1*, *neurogenin2a*, *neurogenin3*, *neurod1* and *dcx* were also increased at 2 dpt, and in some cases also at 6 dpt (Fig. 6e-i). A similar response was observed in astrocytes marker such as *vimentin-a* and *aldh1l1* (Fig. 6j, k). As an indication that the transgene zGFAP::EGFP is not expressed in oligodendrocytes, lower levels of *sox10* and *mbp* were detected in EGFP^+^ than EGFP^−^ cells, in uninjured animals, and these levels were even smaller at 2 dpt, probably as a consequence of the enrichment in the neuronal, and astrocytic lineage (Fig. 6l, m). In line with the analysis depicted above, these results showed that SCI activates NSPCs proliferation, followed by the differentiation of this neural precursor to the neurogenic and/or astrocytic lineage, but not to oligodendrocytes.

To study the function of NSPCs we prepare a transgenic line with the nitroreductase/metronidazol (NTR/MTZ) system in order to specifically ablate these cells [54]. Spinal cord electroporation with a zGFAP::mCherry-NTR construct (see supplementary material, Fig. S7A) followed by incubation with 10 mM MTZ or vehicle (see supplementary material, Fig. S7B) showed a very effective ablation of mCherry^+^ cells once animals were treated with MTZ compared with vehicle treatment (see supplementary material, Fig.S6C-R). Based on these results we prepared the transgenic line Xla.Tg(Dre.gfap:mCherry-Nitroreductase)^Larra^ (see supplementary material, Fig.S5S-U), for short zGFAP::mCherry-NTR.

**Fig. 7:**
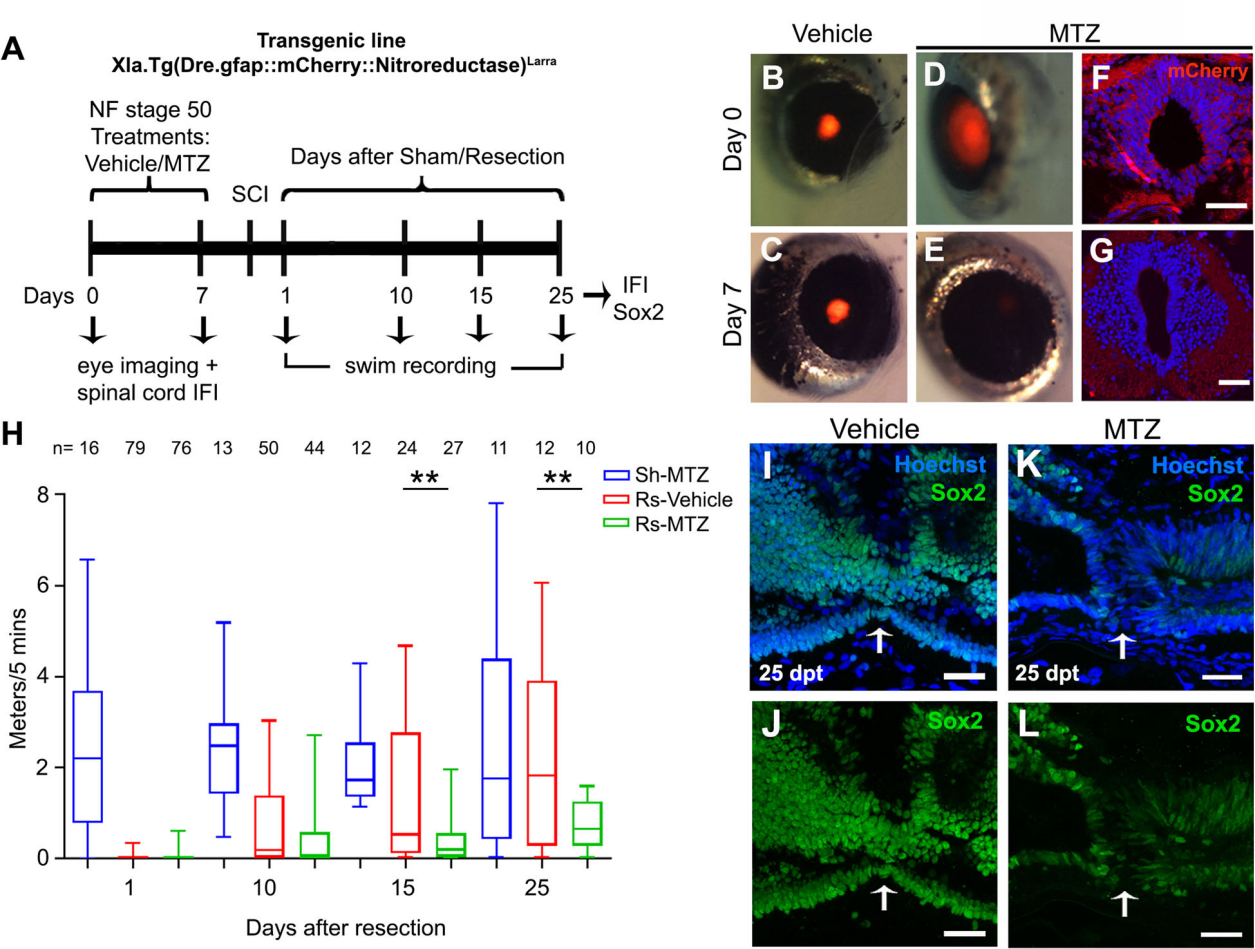
Ablation of NSPCs blocks spinal cord regeneration. **a** Diagram of the treatment of zGFAP::mCherry-NTR transgenic animals with metronidazol (MTZ) or vehicle, followed by spinal cord resection, swimming recording, and histological analysis. **b-e** Eye imaging (**b**, **d**) before treatment, and 7 days after incubation with **c** vehicle or **e** MTZ. **f**, **g** Spinal cord sections showing mCherry expression **f** before, and **g** 7 days after MTZ treatment. **h** Graph of swimming at 1, 10, 15, 25 dpr in sham (Sh) operated animals treated with MTZ (Sh-MTZ, blue boxes), and resected (Rs) animals incubated with vehicle (Rs-Vehicle, red boxes) or MTZ (Rs-MTZ, green boxes). **i-l** Immunofluorescence against Sox2 (green) and nuclei stained with Hoechst of spinal cord sections obtained from animals at 30 dpr from the **i**, **j** Rs-vehicle, and **k**, **l** Rs-MTZ treated groups. Statistics in graph H: ANOVA-one way with Bonferroni post-test, ** *p* < 0.01, *n* = 4 independent biological replicates

We use this line to evaluate the effects of NSPCs ablation in the ability of R-stage animals to regenerate the spinal cord and recover their swimming ability. For this, four groups of animals were considered: i) sham operated animals treated with vehicle or MTZ, and ii) resected animals treated with vehicle or MTZ. Animals were incubated with vehicle or MTZ for 1 week before sham operation or spinal cord transection (Fig. 7a). Efficient ablation of mCherry^+^ cells was attained at 7 days after incubation with MTZ (Fig. 7b-g). After transection, we measured the swimming ability of each group. Treatment with MTZ has no effect on the ability of sham-operated animals to maintain their swimming ability compared to vehicle treated animals (data not shown). Sham operated animals present a 69% survival rate (Fig. 7h, and data not shown). Importantly, at 15 and 25 dpr animals incubated with MTZ showed a diminished swimming ability compared to vehicle treated siblings (Fig. 7h, compare red and green boxes). Resected animals present a survival rate of 30–35% at 20 dpr, and 13–15% survival at 25 dpr, and the death rate is not affected by MTZ treatment indicating that animal death is explained because of the surgery. For this reason higher numbers of resected, than sham animals were used. In addition, we performed Sox2 immunostaining in sections of the spinal cord at 30 dpt, and found that treatment with MTZ, as expected, resulted in a strong reduction of Sox2^+^ cells precluding the proper regeneration of the spinal (Fig. 7i-l). These results indicate that NSPCs cells are necessary for regeneration of the spinal cord of NF stage 50.

## Discussion

### Cellular response in R- and NR-stages

Using electron microscopy and immunofluorescence analysis we found several differences in the cellular response to spinal cord injury between R- and NR-stage. Here, we will summarize our results comparing the cellular response between both stages. One first and notable difference is that the cells lining the central canal at 2 dpt in R-stages, look very healthy and normal. In contrast, central canal cells in NR-stages suffer an important loss of internal organelles, and therefore the main structure of the central canal was heavily affected at early days after injury. This observation raises interesting questions about the mechanism that prevents cells death in R-stages.

Regarding the closure of the spinal cord we observed a very rapid close of the rostral and caudal stumps at the R-stage, a response that is probably very important to maintain the structural homeostasis and organization of the injured spinal cord at the R-stage. Interestingly, it resembles the first step during wound healing, where a platelet plug is initially formed to maintain hemostasis [55]. In agreement with our previous studies [48], the ependymal cells closing the rostral stump were characterized by a high nucleus/cytoplasm ratio and lax chromatin. On the contrary, a cellular closure of the spinal cord canal was just observed after 20 dpt in NR-stage animals.

An active process of cellular extrusion was observed at 2 days after injury in R-stage, in which cells lining the central canal were extruded into the lumen of the central canal. Interestingly, extrusion was mainly detected between 2 and 6 days after the injury. Cell extrusion can act as a regulator from epithelial homeostasis by removing apoptotic cells [56], altering cellular position to regulate development, or to determine cell fate, as happens with neuroblast delamination before initiating the neurogenic divisions in *Drosophila* [57]. In addition, it is also possible to envision that the cells extruded into the central canal can then migrate to the injury site, and participate on spinal cord reconstruction. A process of cellular extrusion was not observed in NR-stages.

The infiltration by immune cells was very limited and controlled in R-stages, besides few red blood cells, some macrophages at the rostral and caudal stumps at 2 dpt were observed. Instead, in NR-stages, cell infiltration constitutes one of the first processes, resulting in an injured site full with red blood cells at 2 dpt, followed by a peak of macrophages phagocyting the red blood cells at the injured site at 6 dpt, similar to what has been reported in rodents [58]. However, at difference with the mammalian counterpart in which macrophages can be found even after 42 days post injury [58], in froglets the macrophages response is limited in time, and after 10 and 20 dpt no more macrophages were found in the injured spinal cords.

Cell proliferation is one of the major hallmarks of the response in the R-stage. Proliferation is already observed at 2 dpt, and is followed by the formation of rosettes structures in the injury site. These structures are composed by cells similar to the ones lining the central canal, and mimics a neural tube like structure. They have cells with adherent junctions, unlike the desmosomes observed in ependymal cells under basal conditions [48], and are surrounded by a basal lamina. These structures are no longer observed with the progress of regeneration, most probably because they collapse in the formation of the new central canal. In contrast, in NR-stages, very low levels of cellular proliferation were detected, but extensive deposition of extracellular matrix was observed in the injury site. In addition, glial processes were already located surrounding the stumps and not crossing the injured site in NR-stages.

Three to four weeks after injury, a complete, although not perfect, regeneration of the spinal cord was observed in R-stage, with a proper reconnection of the central canal, and the restructuration of the CC. A clean central canal with just few extruded cells correlates with the full recovery of the swimming capabilities in R-stage [47]. In contrast, in the NR-stage, axons crossing the injured site are completely absent, and the continuity of the central canal is not restored. At this stage, the injured site is mainly populated by fibroblast-like cells, and filled with ECM components, including collagen and CSPGs. This is confirmed by the increase expression of Vimentin and other glial scar components.

### Role of NSPCs in spinal cord regeneration

We previously described that cells lining the central canal of the spinal cord are possible NSPCs due to the expression of the transcription factor Sox2/3, and their requirement to the regenerative process in R-stages [46, 47]. To understand the identity of these cells, we generated the transgenic line based on the regulatory elements of the GFAP from zebrafish (zGFAP). Although the loss of the *gfap* gene in *Xenopus laevis* has been reported [50], based on the following evidence we concluded that this transgene is expressed in radial glial cells corresponding to NSPCs: i) the cells have a morphology typical of radial glial cells, with an apical process contacting the central canal, and extending a long radial process to contact blood vessels, and the meningeal layer; ii) location, surrounding the central canal in the brain regions such optic tectum, hindbrain and in the spinal cord; iii) timing of expression, EGFP is expressed at early neurulation stages of brain and spinal cord development; iv) RNAseq showed that cells expressing zGFAP::EGFP express the molecular signature of NSPCs, and v) cells expressing EGFP contain intermediate filament, as revealed by the immungold staining. Based on this we propose that the zGFAP::EGFP transgenic line is an adequate resource to study neural stem cell biology.

According to our previous characterization of the cells lining the central canal in the spinal cord of R-and NR-stages, the EGFP^+^ cells correspond to cell types II (dorsal) and III (lateral) in R-stages, and therefore are the highest proliferative cells and the proposed source of cells for repair in case of damage [48]. In NR-stages, the population of EGFP^+^ cells lining the central canal were located only at the dorsal side of the spinal cord, consequently, corresponding to the dorsal radial glial-like cells [48]. However, most of the EGFP^+^ cells at this stage were found in a subependymal layer and express the radial glial marker BLBP and the astrocyte marker GS [59]. This implicates that one of the main source of proliferative cells is reduced in NR-stages, and instead zGFAP::EGFP^+^ cells correspond to a more differentiated type of astroglial cells.

Taking the advantage that the zGFAP::EGFP transgene allows the labeling of the NSPCs, we studied in more detail how they respond to injury. First, as described previously, they responded by massive proliferation [46, 47]. In addition, zGFAP::EGFP^+^ cells extended long EGFP^+^ processes into the injured site, that also expressed the neuronal marker Acetylated tubulin, indicating that these NSPCs differentiate into neurons, confirming the role of neurogenesis in the regenerative process. In addition, we follow the fate of existing or new EGFP^+^ cells taking advantage of the perdurance of EGFP expression. We have found an increase in cells expressing neurogenic factors, or an increase on the expression of these genes in each cell. In particular, we found an increase in *ascl1*, *neurogenin 2a*, *neurogenin 3* and *neurod1* [60], at 2 and 6 dpt. Concomitant with that, we also found an increase in astrocytic markers (*vimentin* and *aldh1l1*) that could be explained because of the self-renovation of radial glial cells, or because some NSPCs differentiate into the astrocytic lineage. On the contrary, no increase of oligodendrocyte markers was detected. This evidence confirms that EGFP^+^ cells act as NSPCs and differentiate into neurons and astrocytes. Mammals also have a small population of NSPCs residing in the ependymal layer lining the central canal of the spinal cord in mice, however, after injury those cells are activated and mostly differentiate into glial cells and oligodendrocytes [10]. The finding that zGFAP::EGFP positive cells are activated to make new neurons and astrocytes, does not preclude the possibility that they can also contribute by providing a permissive substrate or “bridge” for axon regeneration as described as bridge in zebrafish [36, 37].

Furthermore, selective cell ablation of the zGFAP^+^ cells in the CNS in R-stage revealed that after spinal cord injury animals are not able to recover their swim ability. Cell ablation using the Nitroreductase/Metronidazole system has been previously proved to induce specific and selective cell ablation [54] including in a retinal degeneration model in *Xenopus laevis* [61]. This finding confirm that zGFAP^+^ NSPCs cells are necessary for the functional regeneration of the spinal cord in R-stages of *Xenopus laevis.*

### Comparison of spinal cord regeneration with other models of regeneration

Comparing our findings in spinal cord regeneration to other models of regeneration can be of help to identify common and conserved steps in regenerative mechanisms. In addition, such comparisons can provide further clues to identify missing steps, in particular the mechanisms involved in triggering the process of spinal cord regeneration. Among others, the model of tail amputation and regeneration in *Xenopus* contributes with insightful information about the cellular and molecular mechanisms of appendage regeneration in vertebrates [62]. Tail regeneration, including the regeneration of skeletal muscle, the notochord, and the spinal cord, proceeds through a rapid process of wound healing, and initial infiltration (0–24 h post amputation, hpa); followed by proliferation and formation of a blastema-like regenerative bud (1–2 days post amputation, dpa), and an outgrowth phase (2–3 dpa). A key step is the proliferation of tissue specific, and lineage-restricted cellular progenitors that form the regenerative-bud, and give rise to each of its respective tissues, including muscle, spinal cord, and notochord among others [63]. Tail regeneration is very efficient from NF 26–45, and NF48 until tail resorption, but this ability is transiently lost during a refractory period between NF45.47, providing the possibility to compare regenerative and non-regenerative stages.

In previous experiments, we have demonstrated that tail amputation at NF 49 also activates massive proliferation of Sox2/3^+^ cells in the spinal cord, achieving maximal levels of BrdU incorporation at 4–7 dpa. Functional studies showed that these cells are required for tail regeneration [46], indicating a similarity on the cellular mechanism involved in spinal cord regeneration after transection or amputation. Contrary to the expected, recent high-throughput analysis of the response of pax6-expressing neural progenitor cells to tail amputation showed that before proliferation a set of neural progenitors is directly differentiated into neurons through the activation of a neurogenic program during the first 24 hpa. To replenish the regenerated spinal cord of neural progenitors its proliferation is only triggered at 48–72 hpa [64]. This immediate differentiation, without a previous proliferative step, probably allows the formation of new neurons very rapidly supporting a successful regenerative process. These results are compatible with the findings on spinal regeneration reported here, and raise the question about a possible round of neurogenesis even before the activation of NSPCs proliferation in our model of spinal cord transection, a hypothesis that can be tested using the transgenic lines introduced here.

High-throughput analysis, and functional studies in tail regeneration have been a fertile ground to identify early components involved in the activation of the regenerative process, that are conserved among different regenerative models [65, 66]. The generation of reactive oxygen species (ROS) because of the entrance of O_2_ starts at 20 min after tail amputation, and is sustained up to 4 dpa. Early ROS production is absent in the refractory period, and is required to activate diverse signaling pathways, and to allow full tail regeneration in regenerative stages [67]. Since this discovery, a role for ROS as a conserved mechanism among vertebrate and invertebrate regenerative processes has been described [66, 68]. Another response that occurs minutes and hours after amputation is the transient generation of bioelectrical changes particularly at the membrane potential [69]. These electrical currents are also an evolutionary conserved mechanism that is required for tail regeneration. These currents are absent in the refractory period, and are required to activate signaling pathways and proliferation in regenerative stages [70]. Inhibition of ROS production affects bioelectrical signals suggesting that this process is downstream of ROS production [71]. A third component of the evolutionary conserved early regenerative response that has also been discovered in tail regeneration is the rapid recruitment of innate immune cells to the injury site. Although a sustained inflammatory response has been classically described as a process that negatively influences regeneration, more data recently demonstrate that, a proper immune response is necessary for appendage regeneration. The presence of ROS recruits innate immune cells (e.g macrophage and neutrophil-like cells) to the regenerative bud during the first 6 h after amputation [72], and immunosuppression improves tail regeneration in the refractory period, suggesting that an inadequate response is present in this non-regenerative stages [73]. Furthermore, pharmacological blockage of innate immune cells recruitment inhibits limb regeneration in frogs and axolotls [74, 75]. Innate immune cells can also contribute with the production of interleukin 11, which induces proliferation of progenitor cells in the regenerative bud, and is required for tail regeneration [76].

The presence of this early regenerative module, including ROS, bioelectricity, and recruitment of innate immune cells in a diversity of regenerative processes raise the question about its possible role in triggering spinal cord regeneration in our model of spinal cord transection, and particularly in the activation of NSPCs. Here we describe that the number of macrophages detected at 2 dpt is higher in R-stage, but then at 6 dpt high levels of this innate cells are present in the injury site of NR-stage animals, but are no longer present at the R-stage. In line with the findings in tail regeneration, these results suggest that a controlled and transient recruitment of macrophages correlates with spinal cord regeneration, but a sustained and massive infiltration can be detrimental. A comparison of the transcriptome deployed in response to injury in R- and NR-stages showed also higher levels of expression of genes related to inflammation in the NR-stage [49]. Furthermore, we have found that phosphorylation of STAT3, a downstream effector of interleukin-11, is transiently activated, from three to 48 h after injury in NSPCs in R-stage animals. On the contrary, a sustained activation, of up to 6 days, was observed in NR-stages [77]. These findings support the model depicted for tail regeneration, about the requirement of a limited inflammatory response to allow the activation of progenitor cells proliferation, and therefore allowing proper regeneration.

Activation of ROS production, and the generation of electric currents after injury represent good candidates to also be involved in eliciting the activation of NSPCs to allow efficient spinal cord regeneration after transection in R-stage animals. In support of this, ROS play a key role in modulating in vivo and in vitro proliferation, and differentiation of stem cells, including NSPCs [78–80], and recent evidence demonstrated a role for electric currents in regulating the behavior of neural progenitors in the adult mammalian brain [81, 82]. Future experiments should test the possible role of ROS and electric current in the activation of NSPCs in frog spinal cord regeneration, and the different behavior of these components on R- and NR-stages.

Another important process that influences the success or failure of the regenerative process, and is conserved between different regenerative models is the amount and persistence of ECM deposition in the injury site. Here we showed that R- and NR-stage deals differently with ECM deposition. On the one hand, we observe a transient and limited presence of ECM (e.g. collagen) in R-stage, which is persistent and leads to accumulation in the NR-stages. Similarly, R-stage frogs are able to efficiently remove ECM deposition after heart injury, but display a persistent fibrotic deposition in NR-stages that results in the disruption of heart regeneration [83]. Spinal cord regeneration in zebrafish also requires proper deposition of collagen XII to allow axonal growth and regeneration, something that does not occur in non-regenerative animals in which an inhibitory ECM is usually deposited in the injury site [39].

## Conclusions

Cellular response to spinal cord injury dramatically differs between R-stage and NR-stages of *Xenopus laevis*. The regenerative process proceed by the activation NSPCs, followed by its differentiation into neurons and astrocytes. The role of NSPCs in the anatomical recovery of the central canal and spinal cord reconnection is correlated with their requirement for the functional recovery of the R-stage swim abilities. On the contrary, NR-stage respond with the formation of a glial scar and a poor activation of NSPCs. Based on comparisons with other models of regeneration we hypothesize that ROS and electric currents can play a role in the activation of NSPCs, a hypothesis that should be tested in future experiments. Another challenge for the future is to study the possible reactivation of NSPCs in NR-stage animals aiming to promote spinal cord regeneration.

## Material and methods

### Animals

R-stages (NF stage 50) and NR-stage (stage 66) of *Xenopus laevis* were produced by natural mating of wild-type mature male and female frogs obtained from Nasco. Animals husbandry was performed as previously described [40]. All animal procedures were approved by the Committee on Bioethics and Biosafety from the Faculty of Biological Sciences, Pontificia Universidad Católica de Chile (Protocol CBB-004/2013), according to Chilean’s Protection of Animals Act 20380 (2009) and the Guide for the Care and Use of Laboratory Animals (National Research Council, Eighth Edition, 2011).

### Constructs

The CAG-GFP plasmid was obtained from Addgene (Addgene plasmid # 16664). The pEGFP-gfap (Intron1/5′/Exon1-zebrafish) plasmid was a donation from Dr. Pamela Raymond (Addgene, plasmid # 39761), and to facilitate its name was shortened to zGFAP::EGFP. The Osx::mCherry-Nitroreductase plasmid was a donation from Dr. Kenneth Poss (Duke University, USA). The zGFAP::mCherry-Nitroreductase was subcloned by the company Genewiz, by taking the regulatory elements from the zebrafish GFAP [pEGFP-gfap (Intron1/5′/Exon1-zebrafish] and cloned into the Osx::mCherry-Nitroreductase, by replacing the Osx promoter.

### Transgenesis

The zGFAP::EGFP transgenic line was generated from zGFAP::EGFP plasmid. Transgenesis was adapted from [84]. Briefly, eggs were obtained by in vitro fertilization from adult wild type *Xenopus laevis* females. Dejellied one-cell stage embryos were injected with a mixture of linearized plasmid pEGFP-gfap (Intron1/5′/Exon1-zebrafish) or zGFAP::mCherry-Nitroreductase (generated by mixed with sperm nuclei and eggs extract). Embryos were incubated at 18 °C. At NF stage 35 transgenic embryos expressing EGFP or mCherry were selected under a Nikon SMZ-1500 stereoscope. Once embryos reached stage 42, tadpoles were raised at 20–21 °C for 10–12 months until sexual maturation.

### EdU injection and click-iT staining

To identify proliferating cells in the zGFAP::EGFP^+^ cells, R-stage animals (*n* = 3, each stage) received one intracoelomic (i.c.) injection of 50 mg of EdU per gram of body weight at 16 h after 2 dpt or sham control surgery. Animals were anesthetized and fixed by immersion in 4% paraformaldehyde (PFA) as previously described [40]. To analyze EdU labeled cells in zGFAP::EGFP^+^ R-stage, skin and dorsal muscle were dissected for whole mount spinal cord preparation. EdU labeling was performed using the Click-iT EdU Alexa Fluor 555 Imaging Kit (Thermo Fisher Scientific, cat. no. C10338) according to manufacturer’s technical information. Briefly, whole mount spinal cords were permeabilized in PBST 0.5% and then incubated in blocking solution for 30 min at room temperature following by an incubation in the Click-iT EdU reaction cocktail for 1 h. Click it was followed by Immunofluorescence against EGFP and DNA staining with Hoechst (1:10,000). Whole mount spinal cords were mounting with vectashield (Vector Laboratories, H-1000). Confocal z-stack images were taken on an Olympus (Fluoview FV10i) microscope and images were analyzed with Adobe Photoshop (Adobe Systems, San Jose, CA).

### Transmission electron microscopy

For transmission electron microscopy (TEM) analysis, uninjured (ut) R-stage and NR-stage animals and after 2, 6, 10 and 20 after spinal cord injury (*n* = 2/3 each point) were anesthetized and immersed (R-stage) or perfused with 0.83 PBS (NR-stage) followed by incubation in 2% PFA and 2.5% glutaraldehyde (EMS, Hatfield, PA). Spinal cords were micro-dissected and post-fixed overnight in the same fixative. Spinal cords were processes as described before [48]. Briefly, spinal cords at different days post injury were post-fixed in 2% osmium for 2 h, rinsed, dehydrated, and embedded in araldite (Durcupan; Fluka, Buchs, Switzerland). Semi-thin horizontal sections (1.5 mm) were cut with a diamond knife and stained with toluidine blue. To study the cellular response to injury at the different days and stages, ultrathin sections (60–70 nm) were cut with a diamond knife, stained with lead citrate, and examined under a transmission electron microscope (TEM) (Tecnai Spirit G2; FEI, Eindhoven, The Netherlands) by using a digital camera (Morada, Soft Imaging System; Olympus, Tokyo, Japan). Brightness and contrast adjustment of the pictures was performed with Adobe Photoshop (Adobe Systems, San Jose, CA).

### Immunogold staining

For pre-embedding staining, zGFAP::EGFP^+^ R-stage animals (n = 3) were anesthetized and immersed 4% PFA and 0.5% glutaraldehyde. EGFP immunostaining on semi-thin sections was performed as described [48]. Pre-embedding immunogold staining was performed by incubating semi-thin sections in primary antibody (1:200 for anti-EGFP) followed by appropriate colloidal gold-conjugated secondary antibodies (1:50; UltraSmall; Aurion, Wageningen, The Netherlands).

### In vivo time-lapse imaging

The spinal cord of EGFP^+^ animals at R-stage from the transgenic line zGFAP::EGFP were transected, and after 2 dpt an animal was anesthetized in 0,02% of MS-222, the dorsal skin was removed to expose the injured site, and the animal was mounted in low melting agarose 1% in a square chamber with a glass coverslip. In vivo time-lapse imaging was perform on a Olympus (Fluoview FV10i) confocal microscope, z-stack images of the dorsal stump central canal were obtained with a water immersion 60x objective, every 1 h for a total of 7 h. Images were analyzed with Image J and Adobe Photoshop. This experiment was repeated 3 times (data not shown).

### Spinal cord injury

We used two methods to induce spinal cord injury, spinal cord transection or spinal cord resection as described before [40]. Briefly, for spinal cord transection in R-stages, animals were anesthetized, then the skin and dorsal muscles were opened at the mid-thoracic level, and the spinal cord was fully transected with a clean cut at the thoracic level. In NR-stage, after anesthesia, a small incision was made at the skin and dorsal muscle, followed by laminectomy of the dorsal portion of the sixth vertebra, a complete transection through the spinal cord was performed using microdissection scissors. For spinal cord resection, two incisions were made at the spinal cord and a whole section (150–200 μm) was removed in R-stage and NR-stage. Control surgery (sham) were performed at R-stage and NR-stage by an incision at the dorsal skin and muscles but without injured the spinal cord. After surgery, animals were transferred into their tanks with 0.1 x Barth supplemented with antibiotics (penicillin and streptomycin).

### Spinal cord electroporation

R-stage animals were electroporated in the spinal cord as descried before [40]. Briefly, animals were anesthetized in 0.02% MS222, and the plasmid pCAG:EGFP at 2,5 μg/μl (Addgene plasmid # 16664) or zGFAP::EGFP construct at 2,5 μg/μl (Addgene plasmid # 39761) was injected with a glass capillary into the central canal of the spinal cord. Voltage pulses were applied with a Grass SD9 stimulator (GrassTele-factor, USA) across the back using homemade platinum electrodes (5 pulses of 35 V in each polarity, 50 ms pulse length and 200 pps frequency). Animals were transferred into 0.1x Barth containing antibiotics. Screening of EGFP was perform 24 h after electroporation.

### Immunofluorescence

R-stage animals were anesthetized and fixed by immersion in 4% PFA and NR-stage were perfused with 0.83 PBS followed by 4% PFA, the NR-stage spinal cord were post-fixed in the same fixative during overnight. Immunofluorescence was performed as described before [48]. Briefly, spinal cords were embedded in increasing sucrose solutions (5–10–20%), followed by optimal cutting temperature compound (OCT, Tissue Tek®), and frozen in liquid nitrogen. Transversal or sagittal cryosectioned at 10 mm were prepared. Sections were permeabilized in phosphate-buffered saline (PBS; pH 7.4) containing 0.2% Triton X-100 (PBST), then incubated in blocking solution (PBST with 10% goat serum) (blocking solution) for 30 min at room temperature. Sections were incubated with the corresponding primary antibody diluted in blocking solution overnight. Primary antibodies were Acetylated tubulin (1:200; T7451-Sigma); BLBP (1:200, ABN14-EMD Millipore); GS (1:200, MAB302-EMD Millipore); CSPGs (1:100, C8035-Sigma); GFP (1:200, ab6556-Abcam); Fibronectin (1:200, F3648-Sigma); Sox2 (1:200, 4900S-Cell Signaling Technology); Vimentin (1:50, 14 h7-Developmental Studies Hybridoma Bank). Samples were incubated with secondary antibodies conjugated to Alexa Fluor 488 or Alexa Fluor 555 (Molecular Probes, Eugene, OR) at 1:500 in the blocking solution for 2 h at room temperature. Immunofluorescence were followed by DNA staining with Hoechst (1:10.000) and mounting with vectashield (Vector Laboratories, H-1000). Samples were imaged using an Olympus (Fluoview FV10i) confocal microscope and images were analyzed with Adobe Photoshop.

### Acid Fuchsin with Orange-G staining

Collagen analysis was performed in 10 μm horizontal paraffin secions of R-stage (NF stage 50) and NR-stage (NF stage 66) uninjured spinal cord after 6 and 10 days post transection (dpt) in R-stage and after 10 and 20 dpt in NR-stage of three different replicates at each stage and day. For AFOG (Acid Fuchsin with Orange-G) staining, slides were deparaffinized in xylol, followed by rehydration in decreasing ethanol solutions (100% at 40%) and washed in distilled water. Overnight incubation in Bouin’s solution, followed by 30 min wash in running water. Then the slides were incubated in 1% phosphomolybdic acid (10%), wash in running distilled water and staining with AFOG (aniline blue, orange G and acid fuchsin, in a ratio 1: 2: 3 respectively), and incubation in 0.5% glacial acetic acid. Sections were dehydrated with 96 and 100% ethanol, and xylol, slides were mounted with entellan mounting medium and covered with a coverslip. The slides were dry for 1 day at room temperature. Collagen expression was quantified by converting AFOG images into grayscale, defining the scale bar, selecting the blue-stained collagen using thresholding and measuring the thresholded area using imageJ. Quantification was performed in 3 biological replicates at each time-point: uninjured (ui), 6 dpt and 10 dpt of R-stage (NF stage 50) and uninjured (ui), 10 dpt and 20 dpt of NR-stage (NF stage 66).

### Western blot

For western blotting spinal cords from R-stage 50 (*n* = 12) and NR-stage were isolated in uninjured animals (ui) and after 2, 6, 20 and 20 days post injury (dpt). Isolated spinal cords were homogeneized in RIPA lysis buffer with protease inhibitors (benzamidine 1 μM; leupeptin 5 μg/ml; Na_3_VO_4_ 200 μM; phenylmethylsulfonyl fluoride 200 μM and sodium pyrophosphate 200 μM). Western blot was performed as described previously [46, 47]. Proteins were quantified with the protein assay kit (Thermo Scientific) and 20 μg of protein was loaded in each lane. Primary antibodies against Vimentin (1:500, 14 h7-Developmental Studies Hybridoma Bank) and GAPDH (1:10000, EMD-Millipore) were used. Densitometry analysis of Vimentin and GAPDH bands was performed with ImageJ (National Institutes of Health, Bethesda, MD, USA) and in R-stage and NR-stage the Vimentin/GAPDH ratio was normalized to the uninjured (ui) control.

### Cell dissociation and FACS

Spinal cords from EGFP^+^ and EGFP^−^ animals at R-stage from the transgenic line zGFAP::EGFP were dissected from anesthetized animals in MS-222 (*n* = 60), and an enzymatic dissociation in StemPro Accutase (Gibco) in a soft shaking (1–2 speed in a vortex) at room temperature for 30 min. The cell suspension was centrifuged and the cells were resuspended in dissociation buffer (100 μg/mL DNAse I, 5 mM MgCl_2_, 1X HBSS) and the samples keep in the tubes on ice until Fluorescence-activated cell sorting (FACS). For FACS, cells were identified based on size, granularity and EGFP expression. We obtained a 90,1% of viability, based on propidium iodide negative staining. From this cell population, 51% were EGFP^+^ cells and 33% were EGFP^−^ cells and 6,1% of cells between EGFP^+^ and EGFP^−^ cells was discarded from the analysis in order to improve the purification of both cell samples.

### RNA extraction

For the purification of total RNA from EGFP^+^ and EGFP^−^ cells, the commercial kit (RNeasy Mini Kit) was used according to the manufacturer’s. Total RNA was isolated and eluted in water. A treatment with DNase I (QIAGEN) was included. RNA concentration was measured using Nanodrop (Thermo Scientific).

### RNAseq

After cell populations EGFP^+^ and EGFP^−^ were separated using fluorescent activated cell sorter (FACS), total RNA extractions were performed in a single replicate for EGFP^+^ and EGFP^−^ cells and PolyA+ RNAseq libraries were prepared. Later, both libraries were sequenced using Illumina Hiseq4000 platform and obtained a mean of 34,8 (EGFP^−^) and 38,6 (EGFP^+^) million paired-end reads. Sequence quality analysis was performed using FASTQC determining a mean quality score of 27 for reads. Posteriorly, reads were aligned to the *Xenopus laevis* J-strain 9.1 transcriptome (XL_9.1_v1.8.3.2, Xenbase) using Bowtie-RSEM with default parameters [85, 86]. We reached a mapping rate of 70%, comparable between EGFP^+^ and EGFP^−^ cells supporting the robustness of both sequencing results. Differential gene expression among both cell populations was analyzed using DESeq [87] and following the protocol described in its manual for an experiment without replicates, we considered as differentially expressed those genes with fold change ≥2 or ≤ 0,5 and *p*-value ≤0,05. Gene Ontology (GO) enrichment analysis was performed using clusterProfiler [88] and testing for biological process enriched, we considered all GO terms with adjusted p-value ≤0,05. ClusterProfiler was also used for to visualize GO terms enriched in fourth GO level. For to identify EGFP^+^ and EGFP^−^ cells, we compared its expression profiles with a mouse database of different cells types (neurons, astrocytes, oligodendrocyte precursor cells, newly formed oligodendrocytes, myelinating oligodendrocytes, microglia, endothelial cells, and pericytes from mouse cerebral cortex) of the CNS (Ben Barres database [89]). Because this database correspond to a diploid animal model, for to compare we summed counts for homologous gene pairs (L and S genes) and determined differential expression for each pair using DESeq. Posteriorly, we generated a cross database join between homologous gene pairs differentially expressed and genes with fold change ≥4 between cell populations of Barres Database, we choose a high fold change for to highlight differences in expression profiles among cell types. Finally, we evaluated Euclidean distance over FPKM values for to identify the more related cell population to EGFP^+^ cells.

### Real time qPCR

The cDNA from two or three independent biological replicates of EGFP^+^ and EGFP^−^ cells from the transgenic line zGFAP::EGFP at R-stage were prepared from uninjured animals and after 2 and 6 days post transection were synthesized using the M-MLV reverse transcriptase (Promega), and RT-qPCR was performed using Power SYBR Green (Applied Biosystems) by performing three technical replicates on two or three independent biological replicates. The relative expression ratio was calculated as described using *eef1a1* (GenBank: BC043843) as a reference gene. The primers used were *egfp*, *sox2*, *nestin*, *ascl1*, *neurog2a*, *neurog3*, *neurod1*, *dcx*, *vim-a*, *aldh1l1*, *sox10*, *mbp*. For the primers sequence please see supplementary Table 1.

### Cell ablation

For the ablation of the zGFAP::mCherry-Nitroreductase^+^ cells, transgenic animals at R-stage (NF stage 50) were incubated in 10 mM metronidazole (MTZ) prepared in chlorine-free water for 7 days, prior to transection of the spinal cord, and kept protected from light at 21 °C. The medium was changed daily. Control animals were kept in chlorine-free water with and without metronidazole. Cell ablation was evaluated by analysis of mCherry red fluorescence in the eye under fluorescent microscope and spinal cord by confocal imaging.

### Swimming recording

Animals swim at 1, 10, 15 and 25 days post resection or sham control surgery was tracked and recorded as described before [40, 47]. Briefly, an R-stage animal was placed in a 15-cm-diameter Petri dish filled with 100 ml of 0.1x Barth solution. After 5 min of adaptation, a video tracking started for 5 min using the ANY-maze software (Stoelting Co, Wood Dale, IL). The software recorded the trajectory and measured the swimming distance traveled by each animal and the total swimming distance was plotted against the days after injury. Once the test was completed, animal was transferred into their respective tank.

### Cell counting

The number of cells under mitosis was quantified in 4 ultrathin sections from 2 biological replicates at 2 dpt of R-stage, covering a region of 100.000 μm^2^ (500 μm (250 μm rostral and 250 μm caudal) × 200 μm). The number of macrophages and red blood cells were quantified in 4 semi-thin sections from 2 to 3 biological replicates from the TEM analysis at 2 dpt and 6 dpt of R- and NR-stage covering a region of 100.000 μm^2^ (250 μm × 400 μm) including rostral and caudal stumps and the lesion site. Quantification was performed using the cell counter plugin of ImageJ.

To analyze the EdU and EGFP^+^ cells from zGFAP::EGFP transgenic animals at R-stage after 2 dpt and 2 dps (*n* = 3 each group), whole mount spinal cord were imaged under confocal microscope. Z-Stack were analyzed using a cylinder template and cells were counted in the spinal cord and intestine. Double stained cells EdU^+^ and EGFP^+^ cells ere normalized according to the corresponding area in each replicate, the average of the three replicates is shown in each graph. Statistical analyses were performed with ANOVA followed by Tukey’s post hoc test, with results considered significant at *p* < 0.05.

### Statistics

Statistical analyzes were performed with the GraphPad Prism 5 program. For the analysis of swimming capacity, the one-way ANOVA and Bonferroni post-test were used. For the quantification of Red blood cells and Macrofages, for Vimentin western blot and Collagen stained area quantification, as well as, for the analysis of zGFAP::EGFP^+^ cell proliferation, t-Test was performed. Statistical significance was consider as follows: **** (*p* < 0.0001); *** (*p* < 0.001); ** (*p* < 0.01); * (*p* < 0.05).

## Supplementary Information

**Additional file 1: Figure S1.** Cellular response to spinal cord injury in R- and NR-stages. **(A)** Centriolar satellite ultrastructure (arrowheads) in cells surrounding the rostral stump. **(B)** Radial projection of cells lining the central canal (yellow shadow). **(C)** Neutrophil in the injury site at 2 dpt in animals at NF stage 50. **(C-E)** Cells lining a rosette structure at 6 dpt are characterized by a **(D)** basal collagen lamina (blue shadow), **(E)** interdigitations and adherent junctions (arrowheads), and **(F)** intermediate filaments (arrowheads). Graphs of the number of red blood cells/μm^2^ × 10^5^ at **(G)** 2 and 6 dpt in NF stage 50, and **(H)** at 2 and 6 dpt in NF stage 66. Graphs of the number of macrophages/μm^2^ × 10^5^ at **(I)** 2 and 6 dpt in NF stage 50, and **(J)** at 2 and 6 dpt in NF stage 66. t-Test: ** *p* < 0,01; *** *p* < 0,001; **** *p* < 0,0001.

**Additional file 2: Figure S2.** In vivo time-lapse imaging of cells being extruded into the central canal. **(A)** Rostral stump of the transected spinal cord from a zGFAP::EGFP transgenic animal at R-stage 2 dpt. A time-lapses during 7 h for EGFP and transmitted light (T-PMT) z-stack were capture at the following time points: **(B-B′)** 0 min; 60 min **(C-C′)**; 120 min **(D-D′)**; 180 min **(E-E’)**; 240 min **(F-F′)**; 300 min **(G-G’)**; 360 min **(H-H′)**. White and purple arrows point to extrusion events from the cells lining the central canal.

**Additional file 3: Figure S3.** Quantification of Vimentin Western Blot and Collagen AFOG staining. Western blot replicates for Vimentin and GAPDH in uninjured animals (ui), and after 2, 6, 10, 20 dpt in **(A, B)** R-Stage and **(C, D)** NR-Stage. Graphs of the adjusted relative density bands of Vimentin to the GAPDH control and normalized to the uninjured sample (ui) in **(E)** R-stage and **(F)** NR-stage at 2, 6, 10 and 20 days post transection (dpt) spinal cord samples. **(G)** Graph of the adjusted collagen staining area relative to the uninjured (ui) animals at 6, 10 dpt of R-stage and 10, 20 dpt of NR-stage. Red line defined no changes of Vimentin levels or Collagen staining. t-Test: * *p* < 0,05; ** *p* < 0,01; *** *p* < 0,001.

**Additional file 4: Figure S4.** Transgenic line Xla.Tg(Dre.gfap:EGFP)^Larra^. **(A-C)** Three different animals’ electroporated in the spinal cord with the CAG promoter driving the expression of EGFP in central canal cells. **(D-F)** Three different animals electroporated in the spinal cord with the zGFAP::EGFP construct driving specific expression in radial glial like cells in contact with the central canal. **(G-J)** Animals at different developmental stages of the transgenic line Xla.Tg(Dre.gfap:EGFP)^Larra^ showing expression of EGFP in the neural tube at **(G-G’)** NF stage 23; **(H-H′)** NF stage 27; **(I-I′)** NF stage 31 and in the CNS at (**J**-**J’**) NF stage 41. (K-M) Double staining against **(K)** EGFP and **(L)** Sox2 in coronal section of the spinal cord at NF stage 43. Panels **(M)** showed merge image, and panels **(M’, M”)** are magnifications of the dorsal and ventral cells surrounding the central canal.

**Additional file 5: Figure S5.** RNAseq of EGFP^+^ and EGFP^−^ cells isolated from the transgenic line Xla.Tg(Dre.gfap:EGFP)^Larra^. (**A)** Flow chart of RNAseq bioinformatics analysis from EGFP^+^ and EGFP^−^ cells. **(B)** Graph of the Log2 fold change of the differential gene expression between EGFP^+^ cells versus EGFP^−^ cells after FACS and RNAseq. EGFP expression in EGFP^+^ cells (green) is highlighted.

**Additional file 6: Figure S6.** Analysis of EdU^+^ cells in the intestine. **(A-B)** Click-iT staining of EdU^+^ (red) of the intestine in **(A)** sham control animals (2 dps), and at **(B)** 2 dpt. Nuclei were stained with Hoechst (blue). **(C)** Graph of EdU^+^ cells per mm^3^ in the intestine. *n* = 3.

**Additional file 7: Figure S7.** Transgenic line Xla.Tg(Dre.gfap:mCherry-Nitroreductase) allows selective cell ablation. **(A)** Diagram of injection and electroporation of the spinal cord at NF stage 50, indicating volume, concentration and parameters of electroporation. **(B)** Scheme of electroporation of the Dre.gfap:mCherry-Nitroreductase construct and treatment with vehicle or metronidazol (MTZ) at NF stage 50. **(C-R)** mCherry (red) expression in the spinal cord of animal electroporated at **(C-D**; **I-J)** 2 days post electroporation (dpe), before treatment; **(E-F; K-L)** 4 dpe and 2 days post treatment (dtt); **(G-H**; **M-N)** 7 dpe and 5 dtt, and **(O- R)** at 8 dpe and 6 dtt co-stained with Hoechst (blue). **(S)** The construct used to generate the transgenic line Xla.Tg(Dre.gfap:mCherry-Nitroreductase). **(T, U)** mCherry expression in the eye (arrow) and the brain of the transgenic animal at NF stage 42.

**Additional file 8: Supplementary Table 1.** List of genes, ID number and their respective primer-Forward and primer-Reverse used for RT-qPCR analysis.

## Data Availability

RNAseq dataset of EGFP^+^ cells and EGFP^−^ cells is available on NCBI (GSE164204). Xenopus lines, plasmids, and other reagents are available upon request from the corresponding author.

## Abbreviations

AFOG: Acid Fuchsin Orange G
*aldh1l1*: *Aldehyde dehydrogenase1l1*
*ascl1*: *Achaete-scute homolog 1*
BLBP: Brain Lipid Binding Protein
CC: Central canal
CNS: Central nervous system
CSPGs: Chondroitin sulfate proteoglycans
*dcx*: *Doublecortin*
dpe: Days post electroporation
dpr: Days post resection
dpt: Days post transection
dtt: Days post treatment
ECM: Extracellular matrix
EdU: 5-Ethynyl-2′-deoxyuridine
EGFP: Enhanced green fluorescent protein
EM: Electron Microscopy
ERG: Ependymo-radial glia
FACS: Fluorescence-activated cell sorting
FPKM: Fragments per kilobase of exon model per million reads mapped
GFAP: Glial fibrillary acidic protein
GO: Gene Ontology
GS: Glutamine synthase
*mbp*: *Myelin binding protein*
mRNAs: Messenger RNA
MTZ: Metronidazole
*neurog2a*: *Neurogenin2a*
*neurog3*: *Neurogenin3*
NF stage: Nieuwkoop and Faber stage
NR-stages: Non-regenerative stages
NSPCs: Neural Stem Progenitor Cells
NTR/MTZ: Nitroreductase/metronidazole
PBS: Phosphate-buffered saline
PBST: Phosphate-buffered saline with Triton X-100
PFA: Paraformaldehyde
RNAseq: RNA-sequencing
Rs: Resection injury
R-stage: Regenerative stages
SC: Spinal cord
SCI: Spinal cord injury
Sh: Sham operation
Sox2: SRY (sex determining region Y)-box 2
TEM: Transmission electron microscopy
ui: Uninjured
*vim-a*: *Vimentin-a*
*X. laevis*: *Xenopus laevis*
zGFAP: Zebrafish GFAP
zGFAP::EGFP: Construct of zebrafish GFAP regulatory elements driving EGFP and the transgenic line generated in *Xenopus laevis* Xla.Tg(Dre.gfap::EGFP)^Larra^
