# Supplementary figures and images for: Cellular response to spinal cord injury in regenerative and non-regenerative stages in Xenopus laevis
Source: Neural Dev. 2021 Feb 2;16:2. doi: 10.1186/s13064-021-00152-2 (PMC7852093; doi:10.1186/s13064-021-00152-2)

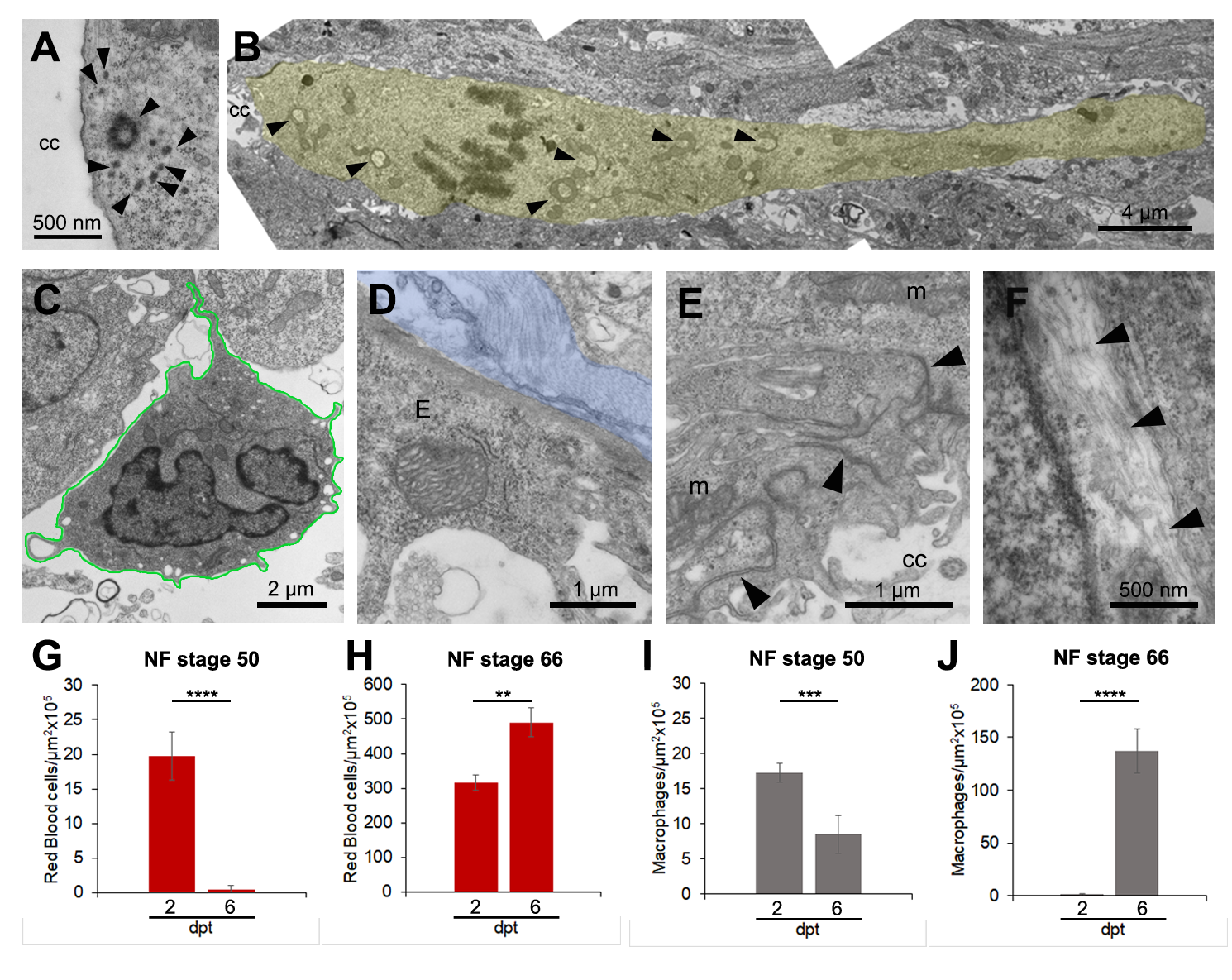

Supplement: Supplementary file 1 — Additional file 1: Figure S1. Cellular response to spinal cord injury in R- and NR-stages. (A) Centriolar satellite ultrastructure (arrowheads) in cells surrounding the rostral stump. (B) Radial projection of cells lining the central canal (yellow shadow). (C) Neutrophil in the injury site at 2 dpt in animals at NF stage 50. (C-E) Cells lining a rosette structure at 6 dpt are characterized by a (D) basal collagen lamina (blue shadow), (E) interdigitations and adherent junctions (arrowheads), and (F) intermediate filaments (arrowheads). Graphs of the number of red blood cells/μm2 × 105 at (G) 2 and 6 dpt in NF stage 50, and (H) at 2 and 6 dpt in NF stage 66. Graphs of the number of macrophages/μm2 × 105 at (I) 2 and 6 dpt in NF stage 50, and (J) at 2 and 6 dpt in NF stage 66. t-Test: ** p < 0,01; *** p < 0,001; **** p < 0,0001. [file 13064_2021_152_MOESM1_ESM.tif]

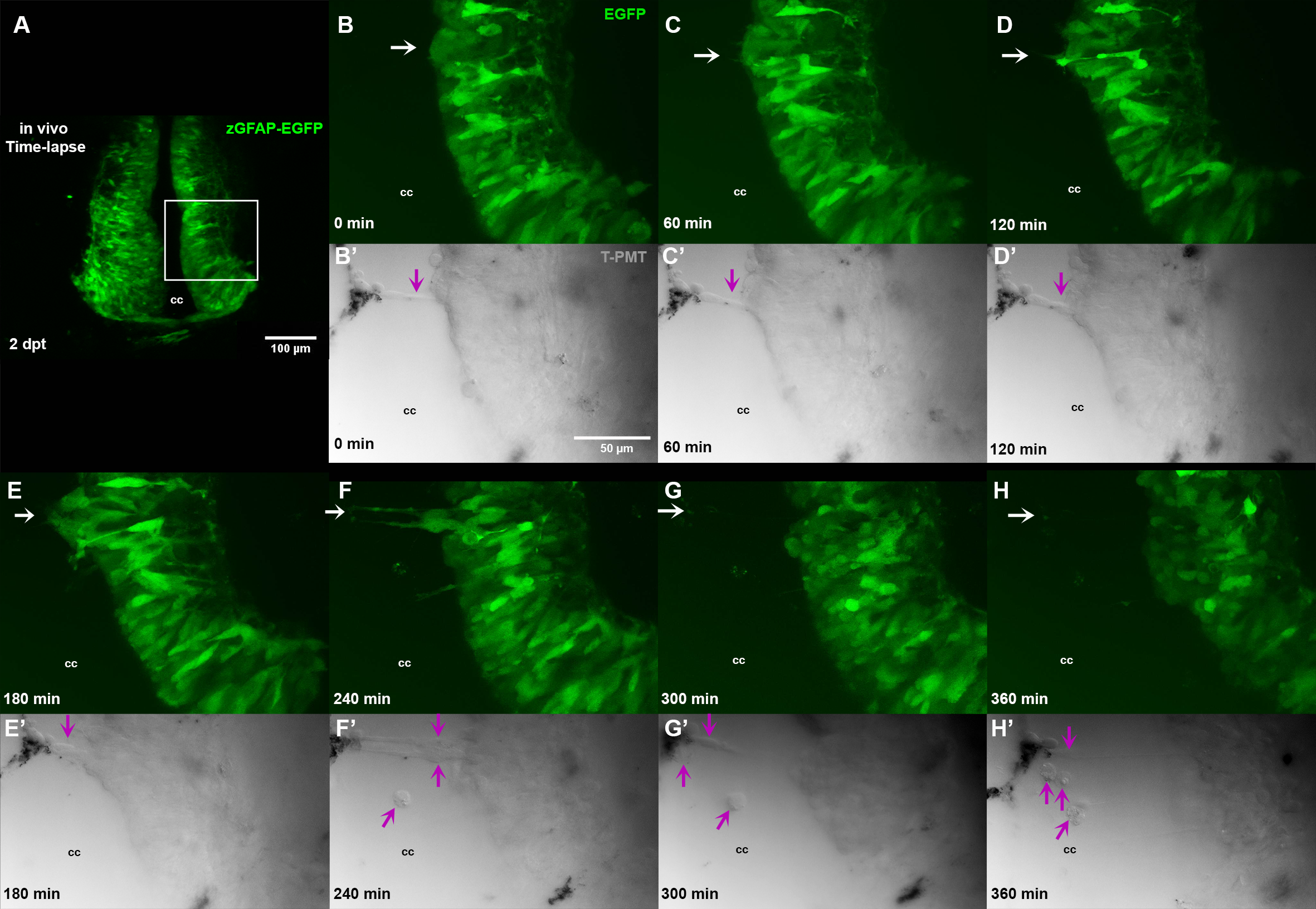

Supplement: Supplementary file 2 — Additional file 2: Figure S2. In vivo time-lapse imaging of cells being extruded into the central canal. (A) Rostral stump of the transected spinal cord from a zGFAP::EGFP transgenic animal at R-stage 2 dpt. A time-lapses during 7 h for EGFP and transmitted light (T-PMT) z-stack were capture at the following time points: (B-B′) 0 min; 60 min (C-C′); 120 min (D-D′); 180 min (E-E’); 240 min (F-F′); 300 min (G-G’); 360 min (H-H′). White and purple arrows point to extrusion events from the cells lining the central canal. [file 13064_2021_152_MOESM2_ESM.tif]

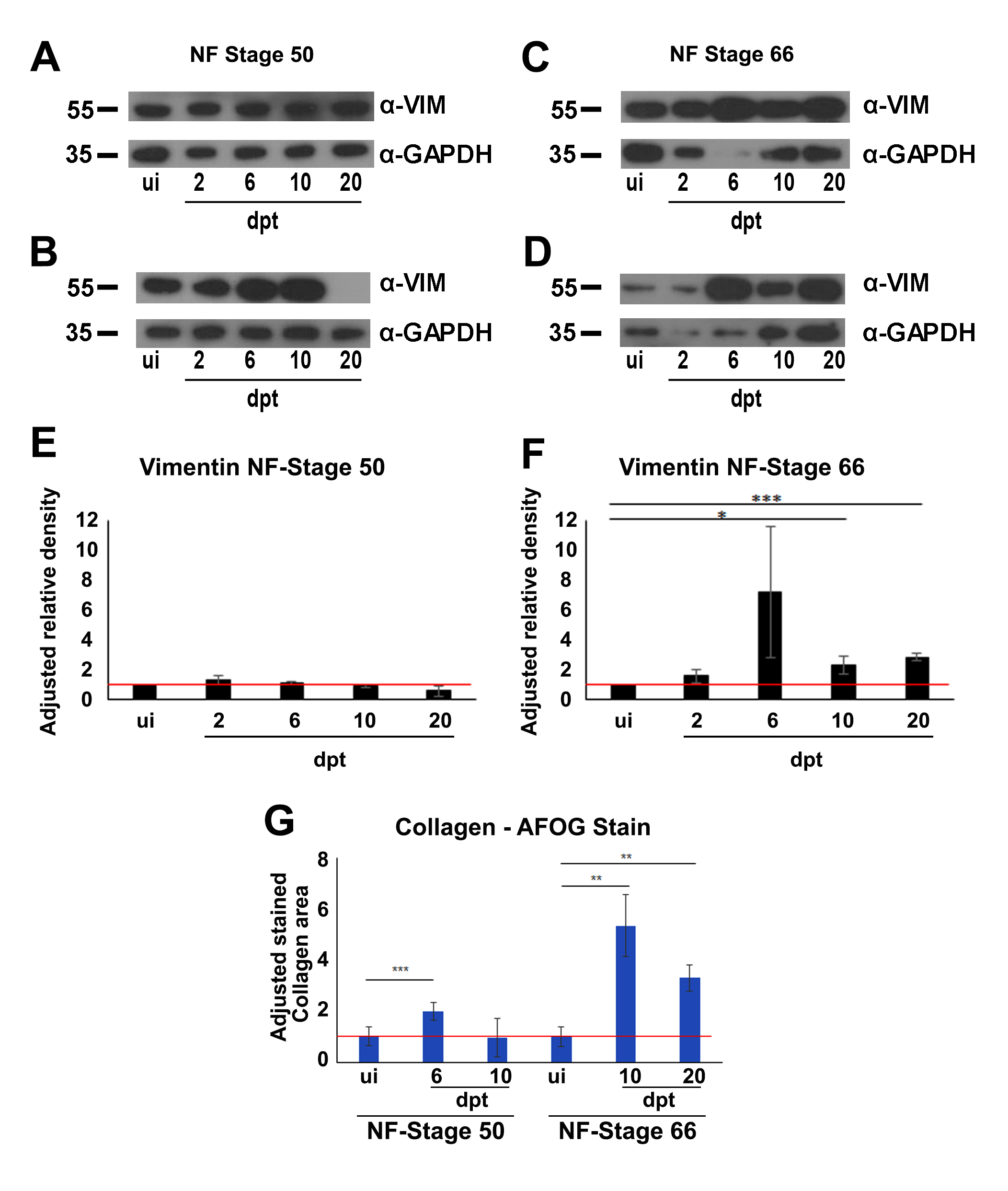

Supplement: Supplementary file 3 — Additional file 3: Figure S3. Quantification of Vimentin Western Blot and Collagen AFOG staining. Western blot replicates for Vimentin and GAPDH in uninjured animals (ui), and after 2, 6, 10, 20 dpt in (A, B) R-Stage and (C, D) NR-Stage. Graphs of the adjusted relative density bands of Vimentin to the GAPDH control and normalized to the uninjured sample (ui) in (E) R-stage and (F) NR-stage at 2, 6, 10 and 20 days post transection (dpt) spinal cord samples. (G) Graph of the adjusted collagen staining area relative to the uninjured (ui) animals at 6, 10 dpt of R-stage and 10, 20 dpt of NR-stage. Red line defined no changes of Vimentin levels or Collagen staining. t-Test: * p < 0,05; ** p < 0,01; *** p < 0,001. [file 13064_2021_152_MOESM3_ESM.tif]

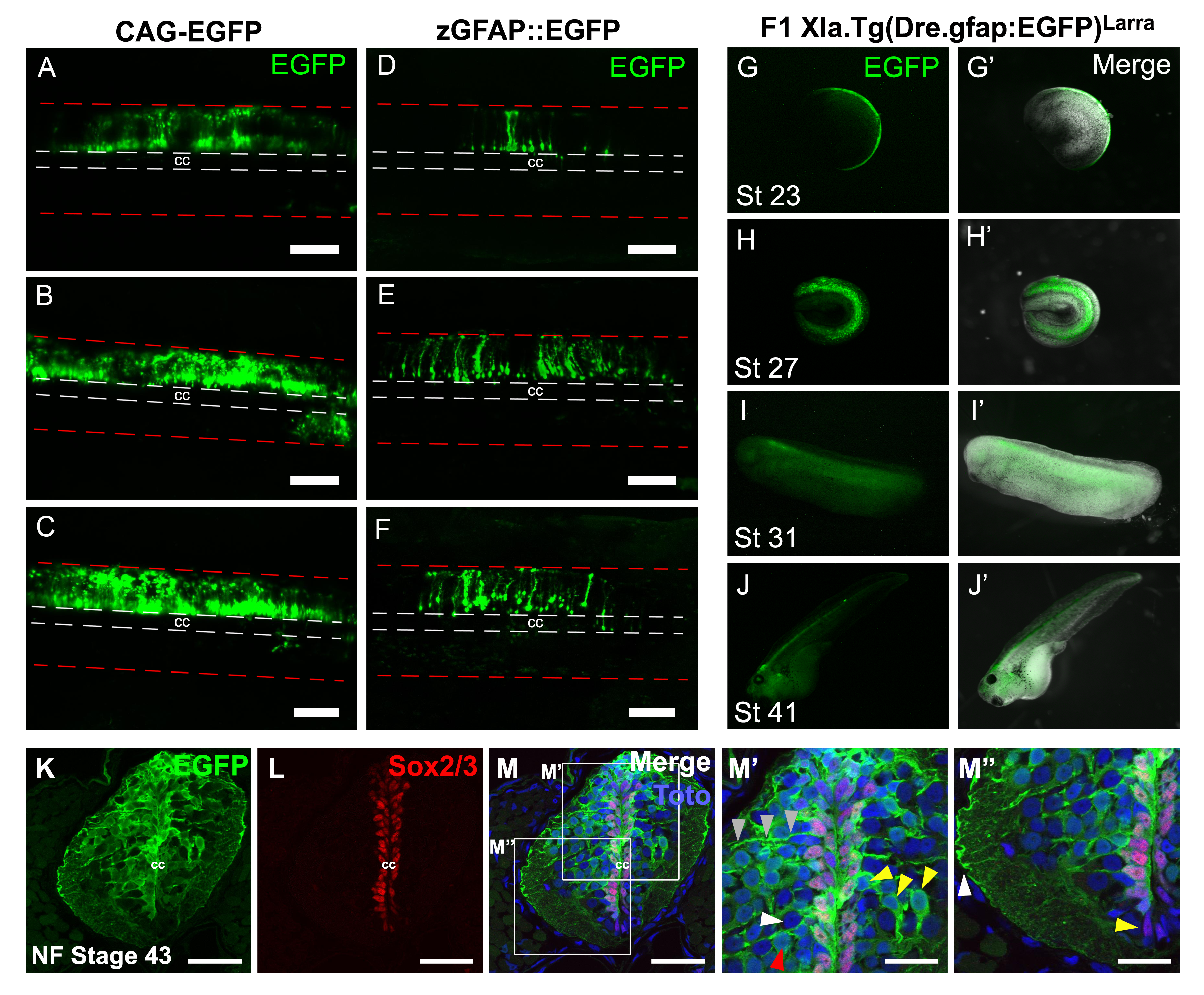

Supplement: Supplementary file 4 — Additional file 4: Figure S4. Transgenic line Xla.Tg(Dre.gfap:EGFP)Larra. (A-C) Three different animals’ electroporated in the spinal cord with the CAG promoter driving the expression of EGFP in central canal cells. (D-F) Three different animals electroporated in the spinal cord with the zGFAP::EGFP construct driving specific expression in radial glial like cells in contact with the central canal. (G-J) Animals at different developmental stages of the transgenic line Xla.Tg(Dre.gfap:EGFP)Larra showing expression of EGFP in the neural tube at (G-G’) NF stage 23; (H-H′) NF stage 27; (I-I′) NF stage 31 and in the CNS at (J-J’) NF stage 41. (K-M) Double staining against (K) EGFP and (L) Sox2 in coronal section of the spinal cord at NF stage 43. Panels (M) showed merge image, and panels (M’, M”) are magnifications of the dorsal and ventral cells surrounding the central canal. [file 13064_2021_152_MOESM4_ESM.tif]

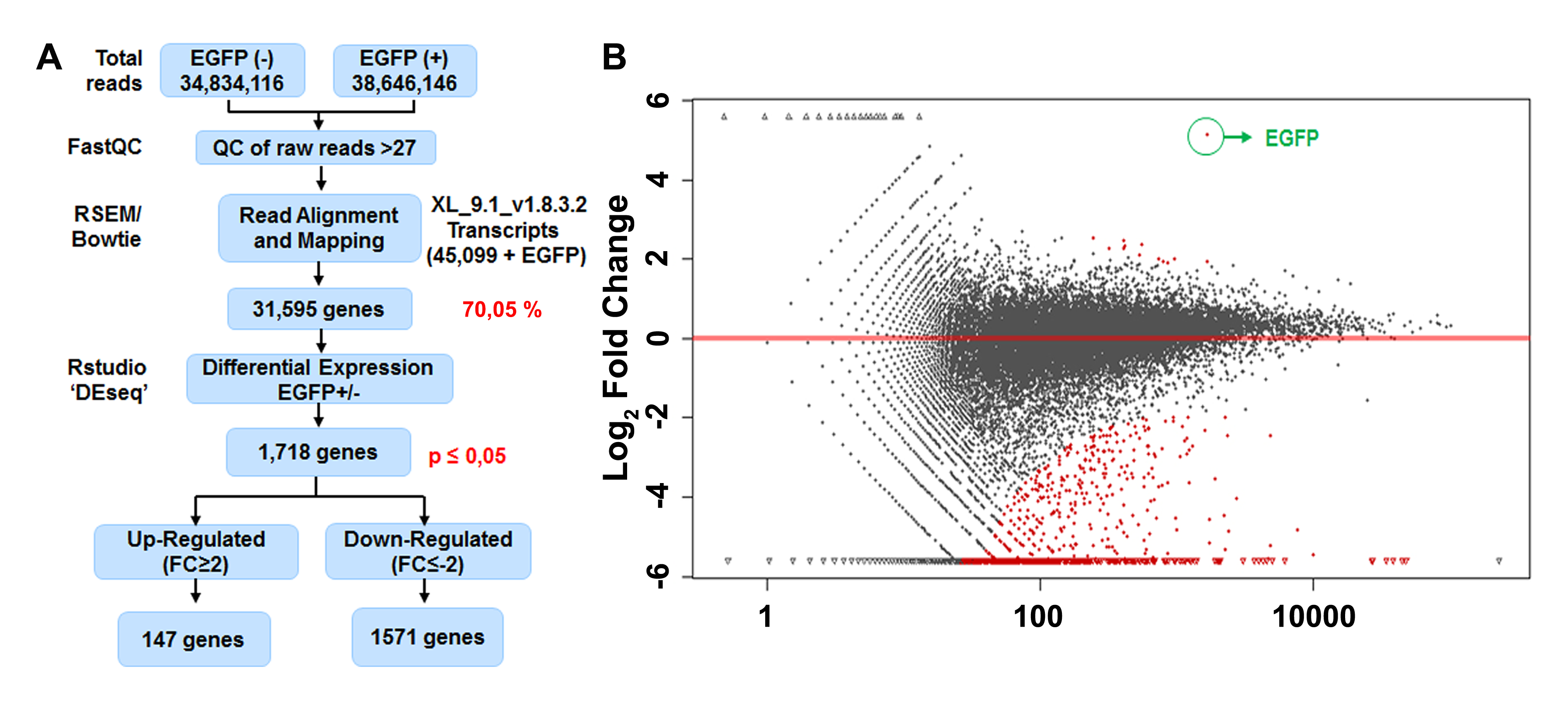

Supplement: Supplementary file 5 — Additional file 5: Figure S5. RNAseq of EGFP+ and EGFP− cells isolated from the transgenic line Xla.Tg(Dre.gfap:EGFP)Larra. (A) Flow chart of RNAseq bioinformatics analysis from EGFP+ and EGFP− cells. (B) Graph of the Log2 fold change of the differential gene expression between EGFP+ cells versus EGFP− cells after FACS and RNAseq. EGFP expression in EGFP+ cells (green) is highlighted. [file 13064_2021_152_MOESM5_ESM.tif]

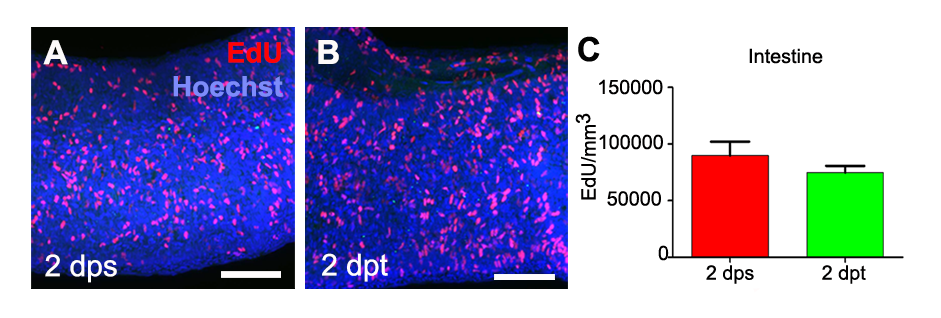

Supplement: Supplementary file 6 — Additional file 6: Figure S6. Analysis of EdU+ cells in the intestine. (A-B) Click-iT staining of EdU+ (red) of the intestine in (A) sham control animals (2 dps), and at (B) 2 dpt. Nuclei were stained with Hoechst (blue). (C) Graph of EdU+ cells per mm3 in the intestine. n = 3. [file 13064_2021_152_MOESM6_ESM.tif]

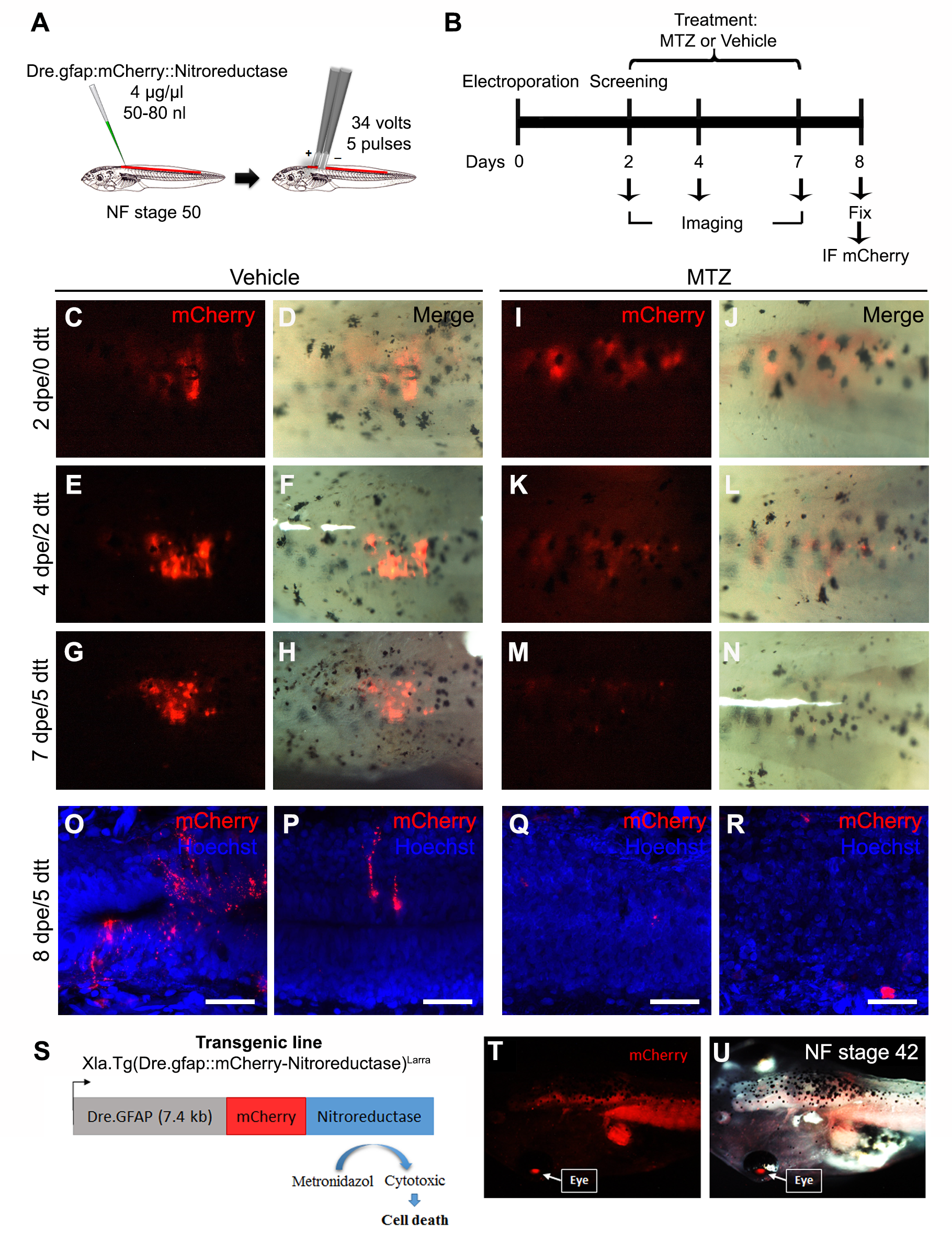

Supplement: Supplementary file 7 — Additional file 7: Figure S7. Transgenic line Xla.Tg(Dre.gfap:mCherry-Nitroreductase) allows selective cell ablation. (A) Diagram of injection and electroporation of the spinal cord at NF stage 50, indicating volume, concentration and parameters of electroporation. (B) Scheme of electroporation of the Dre.gfap:mCherry-Nitroreductase construct and treatment with vehicle or metronidazol (MTZ) at NF stage 50. (C-R) mCherry (red) expression in the spinal cord of animal electroporated at (C-D; I-J) 2 days post electroporation (dpe), before treatment; (E-F; K-L) 4 dpe and 2 days post treatment (dtt); (G-H; M-N) 7 dpe and 5 dtt, and (O- R) at 8 dpe and 6 dtt co-stained with Hoechst (blue). (S) The construct used to generate the transgenic line Xla.Tg(Dre.gfap:mCherry-Nitroreductase). (T, U) mCherry expression in the eye (arrow) and the brain of the transgenic animal at NF stage 42. [file 13064_2021_152_MOESM7_ESM.tif]
