# Supplementary material for: Cellular response to spinal cord injury in regenerative and non-regenerative stages in Xenopus laevis
Source: Neural Dev. 2021 Feb 2;16:2. doi: 10.1186/s13064-021-00152-2 (PMC7852093; doi:10.1186/s13064-021-00152-2)
Supplement: Supplementary file 8 — Additional file 8: Supplementary Table 1. List of genes, ID number and their respective primer-Forward and primer-Reverse used for RT-qPCR analysis. [file 13064_2021_152_MOESM8_ESM.docx]

| **Gene** | **ID number** | **Primer-Forward** | **Primer-Reverse** |
| --- | --- | --- | --- |
| EGFP | 8382257 | CACATGAAGCAGCACGACTT | AGTTCACCTTGATGCCGTTC |
| Sox2 | GQ292559.1 | ACGCTGCCTCTGTCGCACAT | AGAGAGCCACAGTTTGTCCCCC |
| Nestin | NM_001087857.1 | GCCCCTTCTGTCTGGCTGTTGA | GGCTCCACATTTGAGTGGACTCCT |
| Ascl1 | NM_001085778.1 | CCTGTCAGCATGGACAACTG | TTCTGCGGGAAGAAGCAATG |
| Neurog2a | NM_001088333.1 | TATAACCCGACGACTCGACC | CTGTCGCAAGTGACAATGCT |
| Neurog3 | NM_001134785.1 | AACGAGGTCTCCCCTCTCT | TCATTCGCTTGACCCTTTGC |
| NeuroD1 | U28067.1 | GCACTATCCTGCAGCCACTA | CTGGCAATGCACAGTCAGTT |
| Dcx | XM_018232978.1 | CAGGCACTGAGCAATGAAAA | GTCAGCCAACAGAGCATCAA |
| Vim-a | NM_001087438.1 & NM_001087439.1 | AGACTGTGGAGACAAGAGATGG | GTATTACGGTGCTGGCAAAGG |
| Aldh1l1 | NM_001092425.2 | CGTTTTCAAGTTCCCACGTT | TGCCTGGGAAGAATAGATGG |
| Sox10 | NM_001088889.1 | CTCATGCTCACTCCAGTCCA | ACCAATGTCCAGTCGTAGCC |
| Mbp | NM_001090291.1 | GGAAGGGATCAGGAAAGGAG | CAACAGGGTTGTCATCAACG |
| eef1a-1 | NM_001087442.1 | ACGCGTGGGTAAGTGTCCACC | GGCCAGTTGTTGTGGACTTTCCAG |

**Supplementary Table 1**
